# Developmental odontogenic cysts with special focus on the occurrence of multiple cysts and syndromic association: a single-centre cross-sectional study from the Czech Republic

**DOI:** 10.1186/s13023-025-03623-5

**Published:** 2025-03-04

**Authors:** David Szaraz, Albert J. Ksinan, Ctirad Machacek, Petra Borilova Linhartova

**Affiliations:** 1 Clinic of Maxillofacial Surgery, University Hospital Brno, Jihlavska 20, Brno, Czech Republic; 2 Clinic of Maxillofacial Surgery, Faculty of Medicine, Masaryk University, Kamenice 5, Brno, Czech Republic; 3 RECETOX, Faculty of Science, Masaryk University, Kotlarska 2, Brno, Czech Republic; 4 Department of Pathology, University Hospital Brno, Jihlavska 20, Brno, Czech Republic; 5 Department of Pathology, Faculty of Medicine, Masaryk University, Kamenice 5, Brno, Czech Republic; 6 Clinic of Stomatology, Faculty od Medicine, Masaryk University, Pekarska 664/53, Brno, 6250 00 Czech Republic

**Keywords:** Multiple cysts, Non-syndromic, Syndromic, NBCCS, Odontogenic keratocyst, Dentigerous cyst, Orthokeratinizing odontogenic cyst, Developmental odontogenic cyst, Synchronous, Gorlin-Goltz syndrome

## Abstract

**Background:**

This retrospective study aims to evaluate the relative representation of individual types of developmental odontogenic cysts (DOCs), especially from the perspective of syndromic and non-syndromic multiple DOCs in the Czech population. In addition, we also summarize the previous studies on the occurrence of multiple DOCs and provide a literature review of case reports and case series on non-syndromic multiple DOCs, particularly dentigerous cysts (DCs) and odontogenic keratocysts (OKCs).

**Methods:**

The study included histologically confirmed DOCs retrieved between January 1, 2012, and August 8, 2023, at the Clinic of Maxillofacial Surgery, University Hospital Brno, Czech Republic. All specimens were re-classified according to the fifth edition of the World Health Organization Classification of Head and Neck Tumors, 2022. Patients with an uncertain histological diagnosis were excluded from the study.

**Results:**

Of a total of 377 patients, 286 had DCs, 85 OKCs, 5 orthokeratinizing odontogenic cysts (OOCs), 1 botryoid cyst, and 1 calcifying odontogenic cyst. The proportion of patients with multiple DCs in our study (6.6%) was higher than usually reported in the literature. The study also found that 100% of patients with multiple DCs did not exhibit any syndromic associations. On the other hand, 66% of multiple OKCs were associated with the Naevoid Basal Cell Carcinoma Syndrome (NBCCS) and the proportion of OKC patients with NBCCS (7%) was relatively higher than in other studies. Recurrence of OKCs was also significantly associated with NBCCS (*p* < 0.05). Only one patient presented with bilateral OOCs, without any association with a syndrome.

**Conclusion:**

Multiple OKCs are more likely to develop in syndromic patients, while none of the multiple DCs were associated with a syndrome. The incidence of multiple OOCs and other DOCs is extremely rare. Still, we conclude that patients with multiple DOCs should be carefully considered for examination by other specialists to rule out possible syndromic involvement.

**Supplementary Information:**

The online version contains supplementary material available at 10.1186/s13023-025-03623-5.

## Background

Developmental odontogenic cysts (DOCs) arise from tissues that, under physiologic circumstances, give rise to the organs of the tooth. Dentigerous cysts (DCs; 10.6–33% of all orofacial cysts) and odontogenic keratocysts (OKCs; 1.3–21.5% of all orofacial cysts) are the two most common DOCs [1]. Other DOCs, such as the lateral periodontal cyst (LPC), botryoid cyst (BC), orthokeratinized odontogenic cyst (OOC), glandular odontogenic cyst (GOC), and calcifying odontogenic cyst (COC), are much rarer [1] and a vast majority of these cysts are solitary.

In general, multiple DOCs in the same patient unassociated with a syndrome or systemic condition are considered rare or even extremely rare [2–5]. Multiple DCs are reported to usually arise as a part of clinical manifestations of syndromes such as mucopolysaccharidoses (MPS, Hurler syndrome, Hunter syndrome, Morquio syndrome, Maroteaux-Lamy syndrome) [6–11], cleidocranial dysplasia [12], Gardner syndrome (a form of familial adenomatous polyposis) [13], Klippel Feil syndrome [14], or branchio-skeleto-genital syndrome (Elsahy-Waters syndrome) [15–18]. Also, bilateral DCs were observed in two patients with achondroplasia [19, 20]. Multiple OKC phenotype is associated with the Gorlin-Goltz syndrome, also called the Naevoid Basal Cell Carcinoma Syndrome (NBCCS). The most common clinical features of the syndrome include multiple basal cell carcinomas, palmar and plantar pitting, and calcification of the falx cerebri [1]. Recently, some of these features were also observed in a patient with multiple DCs [21]. Besides the NBCCS, multiple OKCs were occasionally observed in other syndromes as well, such as in Ehlers-Danlos syndrome [22, 23], oral-facial-digital syndrome, also known as Papillon-Leage and Psaume syndrome [24], mental-retardation overgrowth syndrome, also known as Simpson-Golabi-Behmel syndrome [25, 26], and in Noonan syndrome [27]. Even a case simultaneously presenting an OKC and bilateral DC was reported [28]. Other multiple DOCs, such as multiple OOCs [29, 30], BCs/LPCs [31], COCs [32, 33], and GOCs [34, 35] are very rare and, to the best of our knowledge, are not associated with any syndrome.

While the relation of multiple DOCs with NBCCS is well established [36, 37], it is less explored in other syndromes mentioned above. Oral manifestations such as multiple DOCs, however, might be the first sign of a syndrome; therefore, it is crucial to gain a good understanding of the prevalence of multiple DOCs in a given disease. In NBCCS, for example, multiple OKCs might be not only the first but also the only clinically apparent manifestation in children [38], although OKCs are often only solitary [39, 40]. This has led to a proposal of new criteria for diagnosing NBCCS early on, facilitating the initiation of preventive measures to avoid or treat other manifestations in time, such as basal cell carcinomas [41]. It is worth noting that OKCs are not the sole lesions that might appear in NBCCS patients. Several cases of ameloblastomas or ameloblastic changes within a cyst have been reported in patients with NBCSS as well, which may complicate the diagnostic process [42].

MPS has usually an early onset of clinical signs. However, just like with NBCCS [43], there are exceptions to the rule due to attenuated forms [44–46]. Detection of multiple DOCs may aid early diagnosis of this disease, which is crucial since prompt treatment may significantly mitigate progression [47]. These examples show the pivotal role of detecting multiple DOCs as warning signs of a hidden disease, and, therefore, the dentist can be the first specialist involved in the diagnostic process. This, however, requires a good understanding of the prevalence and relationship of multiple DOCs with various syndromes. In some syndromes, we lack substantial data as only isolated case reports and small case series are published.

In view of the lack of data on multiple DOCs, this retrospective study aimed to shed more light on their prevalence and to explore their possible relationship to syndromes by (i) evaluating the distribution of various types of DOCs in patients presenting with these cysts over the last ten years in a large university hospital in the Czech Republic serving a population of approx. 1.7 mil and (ii) by investigating the occurrence of multiple DOCs in syndromic and non-syndromic patients in this patient group.

## Materials and methods

Our epidemiological retrospective study was conducted by retrieving records for all patients treated for a jaw cyst from January 1, 2012, till August 8, 2023, at the Clinic of Maxillofacial Surgery, University Hospital Brno, Czech Republic. In this cross-sectional single-centre study, the patients were selected based on the electronic hospital record system; records of all patients with the diagnosis K090 according to the International Classification of Diseases (Developmental odontogenic cysts) were retrieved. Of those, only patients with histologically confirmed DOCs were included in this monocentric study. All applicable data were extracted from patient records, patients with a syndrome were distinguished from non-syndromic patients. Data extraction was performed by trained reseachers using a standardized data extraction form to ensure consistency across all cases. To further ensure data accuracy, 10% of the anonymized extracted records were independently reviewed by a second researcher. NBCCS was determined based on clinical diagnostic criteria as described by Kimonis et al. (1997) [48]. NBCCS in these patients was subsequently confirmed by genetic testing. In other multiple DOCs, a syndrome was ruled out based on patient history and clinical examination.

All histological hematoxilin-eosin stained specimens were re-examined and re-classified according to the fifth edition of the World Health Organization Classification of Head and Neck Tumors, 2022, by an experienced pathologist specialized in oral pathology [49]. Patients with an uncertain histological diagnosis were excluded from the study. The relations between DCs and particular impacted teeth were also evaluated. Other DOCs were subclassified according to their location – posterior vs. anterior mandible or maxilla. Further, multiple DOCs in the same patient were classified separately within the particular cyst group.

Chi-square tests were used to compare the proportions of categorical variables. After checking the assumptions for normality, t-test was used to compare differences in mean age across groups. For the subsample of patients with multiple cysts, non-parametric tests were used: a Wilcoxon rank-sum test for comparing mean age across diagnoses, and Fisher’s exact test for comparing the distribution of diagnoses by sex and OKC recurrence with NBCCS. All analyses were computed in R 4.3.2.

The research was conducted in accordance with the principles of the Declaration of Helsinki; the study was waived by the Ethics Committee of the University Hospital Brno in view of the retrospective character of the study (Decision No. 08-120619/EK).

## Results

### Types of cysts

In total, 377 patients with DOCs were identified in our records and included in the study. Among these, 286 were patients with DCs (308 cysts), 85 with OKCs (107 cysts), 4 with OCCs (5 cysts), 1 patient with a COC, and 1 patient with a BC (Supplementary Table S1). All cysts were treated by extirpation, except for one OKC treated by marsupialization only and four OKCs treated by extirpation after a previous marsupialization. Recurrence was observed only in 14 patients with OKC.

Most DCs arose around the impacted wisdom teeth of the mandible (*n* = 258), followed by the maxillary wisdom teeth (*n* = 23), canines (*n* = 12), second premolars (*n* = 7), second molars (*n* = 4), and supernumerary teeth (*n* = 4) (Supplementary Table S1).

**Fig. 1:**
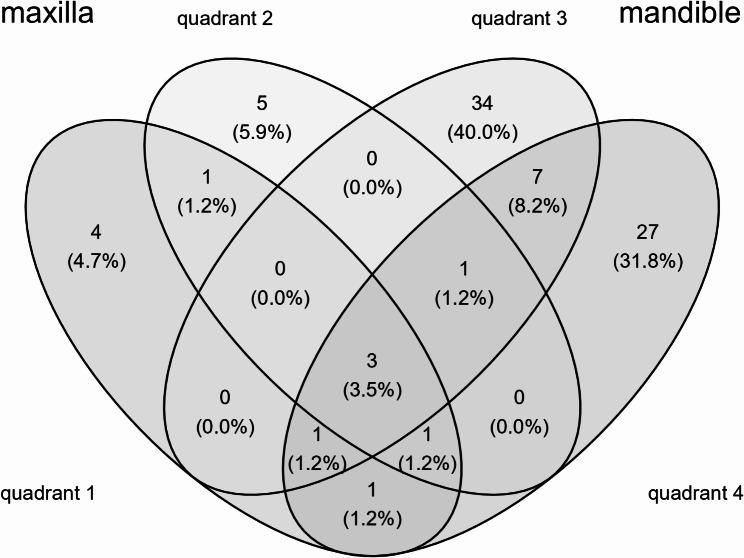
Venn diagram demonstrating the predominant odontogenic keratocyst occurrence in the mandible (quadrant 3 and 4)

Solitary OKCs (73 patients) were more than seven times more common in the mandible than in the maxilla (64:9) (Fig. 1). In the mandible (64 cases), the ratio between the frontal and the distal areas was 1:11, with four cysts affecting both regions. In the maxilla (9 cases), the ratio was much lower (1:2). Among the 76 cases, OKCs were associated with an impacted tooth in 22 patients (a total of 36 cysts), mostly with the wisdom teeth in the mandible.

### Demographic data

The mean age of the patients was 47.32 years (standard deviation, *SD* = 17.78) with an age range of 7–91 years. Of those, 66% were male patients.

**Fig. 2:**
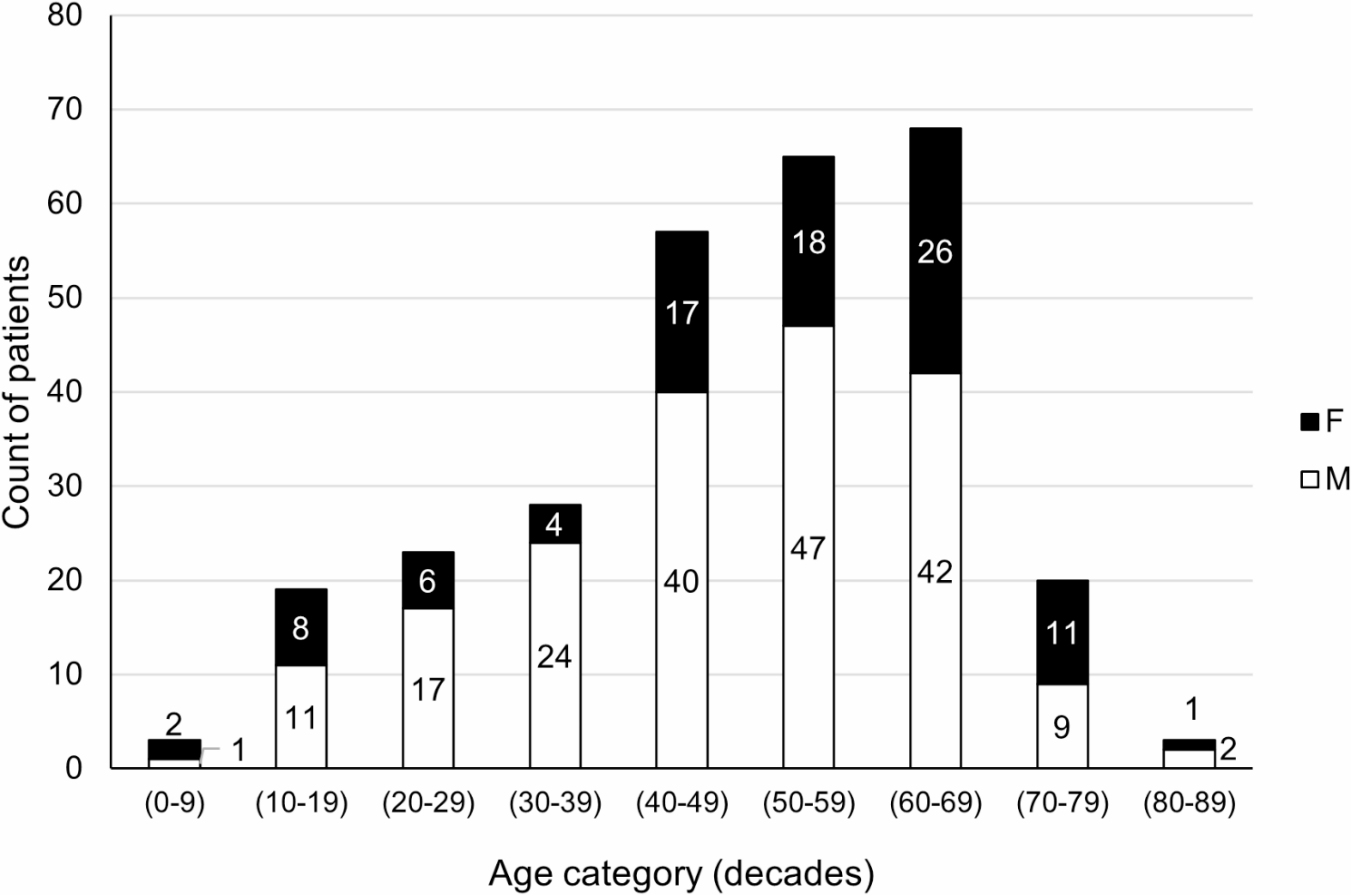
Age and sex distribution of 286 patients with dentigerous cysts F – Female, M – Male

DCs were diagnosed mostly in patients in their forties to sixties (Fig. 2). The mean age of patients with DCs was 49.22 years (*SD* = 17.05).

**Fig. 3:**
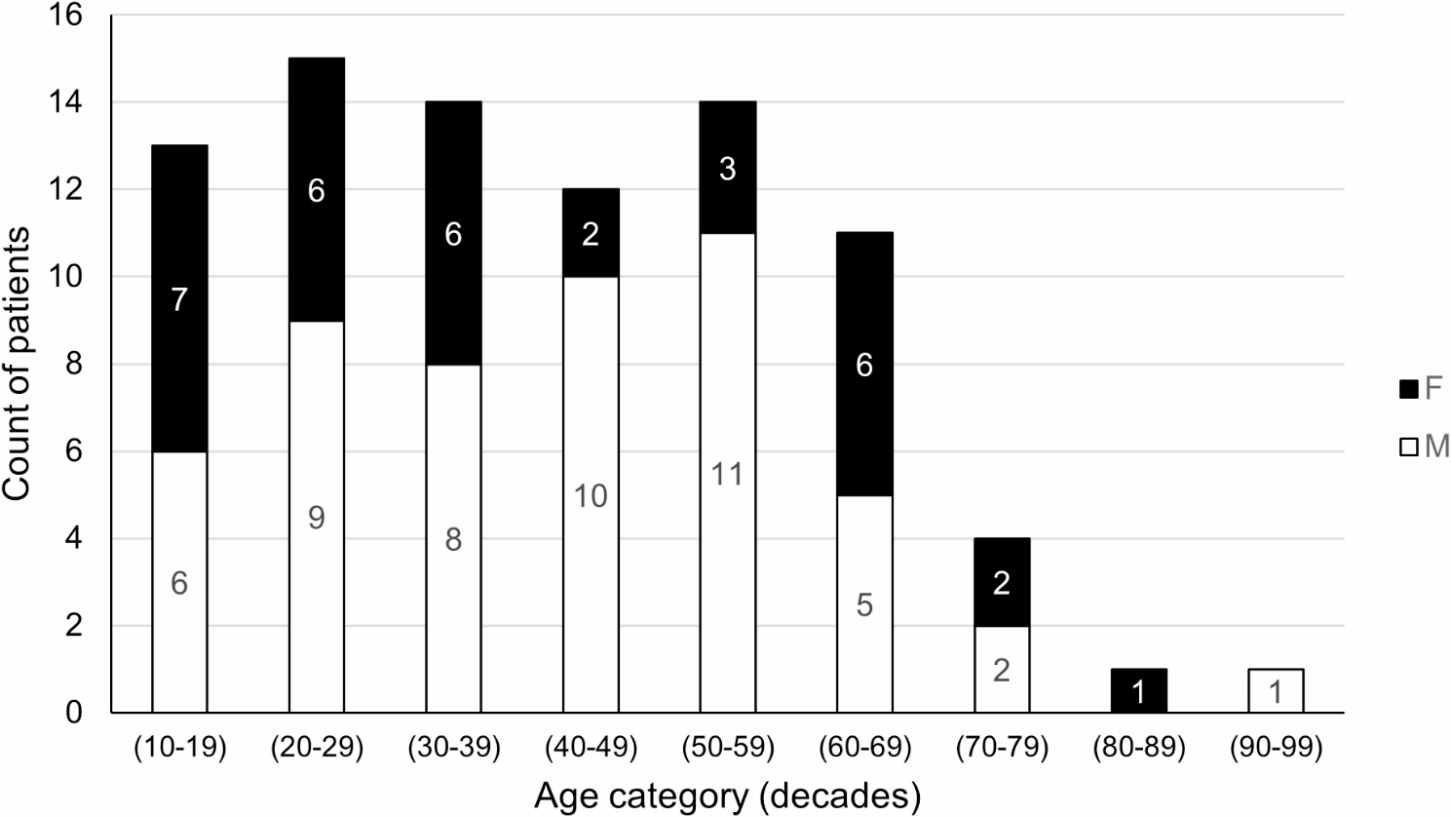
Age and sex distribution of 85 patients with odontogenic keratocysts F – Female, M – Male

Of all OKC patients, 41% were female. Compared to DCs, OKCs were much more prevalent in younger patients, with a mean age of 42 years (Fig. 3). This is significantly lower than in the DC group (*M*_DC_ = 49.22 vs. *M*_OKC_ = 41.33, *t*(126.1) = 3.42, *p* < 0.001, 95% CI [3.32, 12.46]).

### Multiple cysts, association with syndromes

**Fig. 4:**
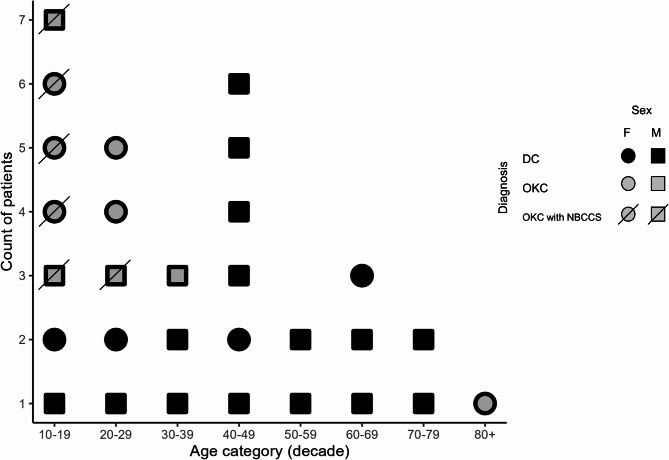
Patients with multiple developmental odontogenic cysts by type, sex, and association with Naevoid Basal Cell Carcinoma Syndrome DC – dentigerous cyst, OKC – odontogenic keratocyst, NBCCS – Naevoid Basal Cell Carcinoma Syndrome

Multiple DOCs were found in the DC, OKC, and OOC groups (Supplementary Table S1). The median age of patients with multiple OKCs was significantly lower than in patients with multiple DCs (median age: DC = 45 years, OKC = 25 years, *W* = 149.5, *p* = 0.013, 95% CI [7, 36]). The majority of patients with multiple OKCs were female (4 men vs. 6 women), while the opposite was observed in patients with multiple DCs (15 men vs. 4 female, Fig. 4, *p* = 0.050 based on Fisher’s exact test). Both OKCs and DCs occurred in the mandible more commonly than in the maxilla (Fisher’s exact test, *p* = 0.003).

### Multiple DCs

**Fig. 5:**
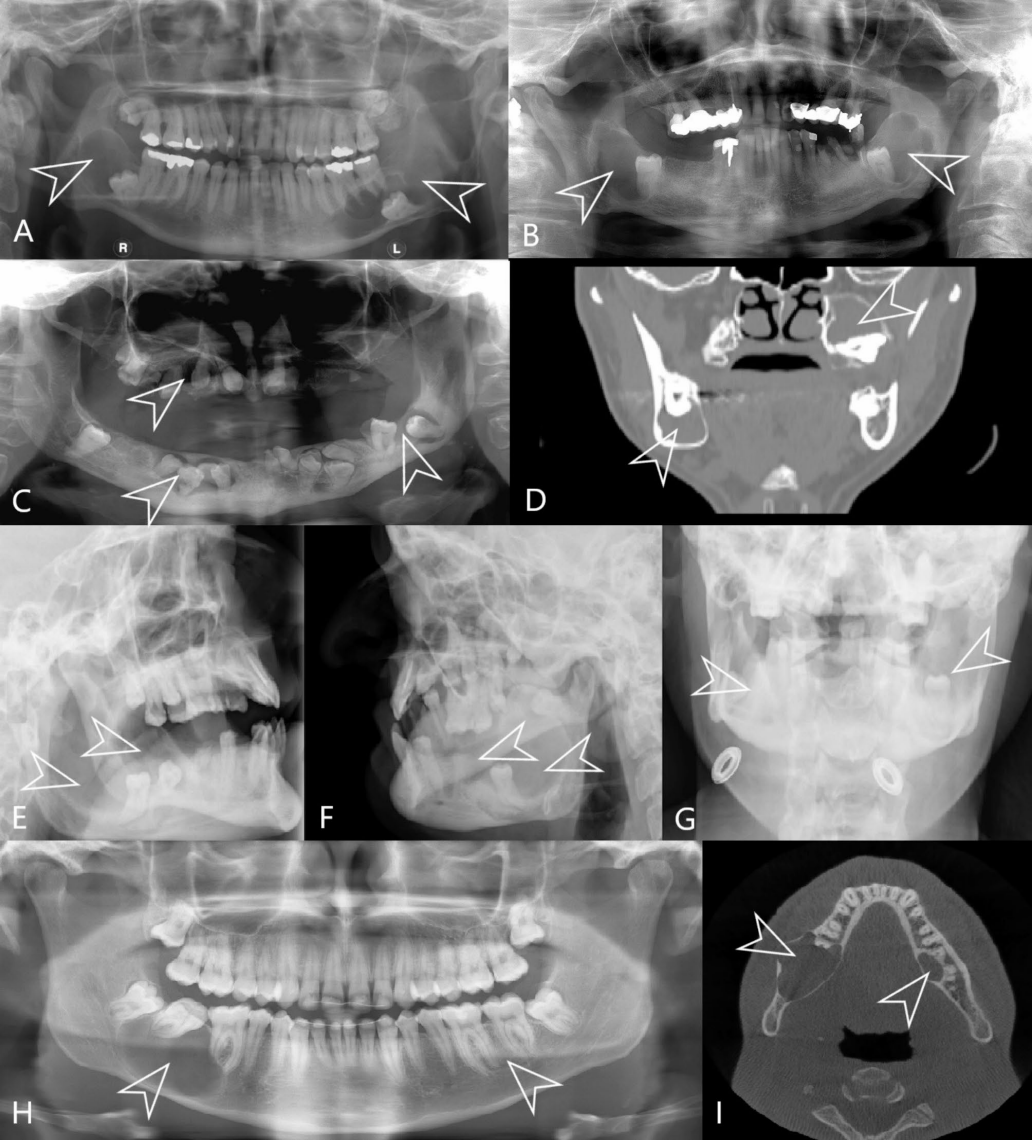
Examples of multiple non-syndromic developmental odontogenic cysts. Locations of the cysts are marked with white arrows. **A**, **B** – Panoramic X-rays of bilateral dentigerous cysts (DCs), **C** – Panoramic X-ray of multiple non-syndromic DCs, **D** - Computed tomography of non-syndromic DCs of the maxilla and mandible. **E**, **F**, **G** – X-rays of the head of a patient with bilateral non-syndromic odontogenic keratocysts. **H**, **I** – Panoramic X-ray and Cone-beam computed tomography of a patient with bilateral orthokeratinizing odontogenic cysts

**Fig. 6:**
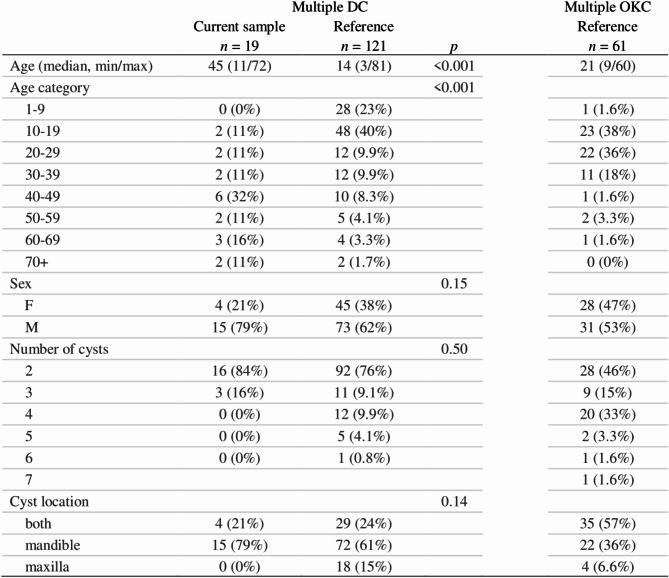
Summary of data of patients with multiple DCs included in the study, of patients with multiple DCs included in Supplementary Table S2, and of patients with multiple OKCs included in Supplementary Table S3 DC – dentigerous cyst, OKC – odontogenic keratocyst

Among the 19 patients with multiple DCs with a total of 41 lesions, two cysts were observed in 16 patients. They were mostly bilateral and associated with mandibular third molars (Fig. 5A, B). There was only one exception in a patient with one DC associated with a third mandibular molar but the other cyst was associated with a maxillary third molar (Fig. 5C). Three patients developed three synchronous cysts, in one case with multiple impacted teeth (Fig. 5D). None of these patients had a syndrome associated with craniofacial dysmorphisms and/or jaw cysts or any other syndrome. The mean age of patients with multiple DCs was 45 years, which is significantly higher compared to the literature (Fig. 6). This difference, however, might be explained by the fact that our clinic primarily focuses on adult patients (despite the fact that we also treat pediatric patients with larger lesions, children with smaller lesions can be treated elsewhere).

### Multiple OKCs

Although only nine patients had multiple OKCs, the prevalence of multiple cysts in the OKC group was proportionally higher compared to the DC group. However, in 6 of these patients (3 women and 3 men), the multiple OKC occurrence was associated with NBCCS and only three patients (2 women and 1 man) were non-syndromic (Fig. 4). All 6 NBCCS patients met two major diagnostic criteria (the presence of multiple OKCs and basal cell carcinomas), in addition to which the syndrome was confirmed by genetic testing. Also, the recurrence of OKCs was significantly associated with NBCCS (compared to multiple non-syndromic and sporadic OKCs; Fisher’s exact test, *p* = 0.021; see Supplementary Table S1). The mean age of the NBCCS group was 14.67 (*SD* = 4.50), while the mean age of the non-syndromic group was 43.35 (*SD* = 18.32). One patient from the NBCCS group had OKCs in two quadrants, three patients had OKCs in three quadrants, and two patients had OKCs in all four quadrants. One of the non-syndromic patients had three metachronous OKCs with one jaw cyst arising in the right mandible, another cyst in the left mandible, and, at a later time, he developed a third one in the left maxilla. In the other two non-syndromic patients, the lesions were synchronous and associated with impacted wisdom teeth. One of the patients had severe mental retardation with an incidental finding on X-rays of the head (Fig. 5E, F, G); however, no known syndrome could be associated with this disorder. Nevertheless, all patients with multiple OKCs were referred for further counseling to rule out a possible syndrome involvement.

### Multiple OOCs

Of the four patients with OOCs (all male, *M*_age_ = 36.5 years [*SD* = 13.03]), only one presented with bilateral cyst development. This generally healthy 20-year-old patient without any syndrome or systemic disorder developed one cyst in the area of tooth 48 and the other on the lingual side of teeth 36 and 37 (Fig. 5H, I). No recurrence was observed after extirpation.

## Discussion

### Demographic data

The results of this study are noteworthy for several reasons. The patients in the DC group were older than usually reported (see below) – most were diagnosed in the age range of 40 to 69. Some studies reported a peak in the second to the fourth decade [50–52]. Other studies reported the peak in the 5th and 6th decades [53–55], but in the 7th decade, a decrease was expected based on those results. In our population, however, the opposite results were found – in the 7th decade, the incidence did not decrease; it was even, albeit negligibly, higher than in the previous ones. Further observations like the male-to-female ratio and the relation to the particular impacted teeth were in line with previous studies [1]. Regarding the OKC group, the demographic data are consistent with the results of previous studies – the majority of patients were male and most of the patients were in the second, third, and fourth decades of their lives [1]. The location of the occurrence (mostly the lower jaw in the distal area) was also in line with previous studies.

### Multiple DCs

In our study sample, the proportion of patients with multiple DCs (6.6%) was higher than what is usually reported in the literature (Table 1). The only two studies reporting higher numbers of patients with multiple DCs are those by Noujem and Nasr (2021) [52] and Fickling (1968) [56], with an exceptionally high rate of 22.9% and 20.9% respectively. Where the proportion of the total number of multiple cysts is concerned, it was 13.3% in our cohort. Again, two studies reported a higher proportion of multiple cysts [52, 57]. However, it has to be emphasized that the number of patients with multiple cysts is probably a better indicator of their prevalence as the number of DOCs can vary among patients (some may have two, some three, and some even more DCs).

**Table 1:** Prevalence of multiple dentigerous cysts (DCs) in different countries

Region	Total number of				Reference
	patients with a DC	patients with multiple DCs	DCs	multiple DCs	
UK	N/A	7	67	14 (20.9%)	Fickling 1965 [56]
Chile	N/A	N/A	546	61 (11.0%)	Ochsenius et al. 2007 [50]
British Columbia, Canada	2029	51 (2.5%)	2082	N/A	Zhang et al. 2010 [51]
Taiwan	332	6 (1.8%)	338	12 (3.6%)	Lin et al. 2013 [77]
Turkey	N/A	3	114	N/A	Karabas et al. 2020 [55]
Lebanon	109	25 (22.9%)	137	53 (38.7%)	Noujeim and Nasr 2021 [52]
Massachusetts, USA	198	N/A	218	37 (17.0%)	Caruso et al. 2022 [57]
Czech Republic	286	19 (6.6%)	308	41 (13.3%)	Current study

N/A – non-applicable/specified, or unknown

It should be noted that the practice and timing of extracting the impacted wisdom teeth will certainly affect the prevalence of DCs, including multiple ones. In countries where people are more encouraged to get their impacted teeth extracted at a younger age, the prevalence of both solitary and multiple DCs will be lower [51]. This might cause the false impression that multiple DCs are not very common in such countries.

Bilateral or multiple DCs are usually reported to occur predominantly in patients with syndromes associated with impacted teeth or supernumerary teeth [1, 3–5, 14]. While this seems to be true for MPS [7–10, 47], we have not found any literature on the prevalence of multiple DCs in the other syndromes associated with these cysts, such as cleidocranial dysplasia or Gardner syndrome. A radiographic study of 40 patients with cleidocranial dysplasia did not report such findings on panoramic X-rays [58], neither did case reports or studies on oral manifestations of familial adenomatous polyposis [59–64].

Where MPS patients are concerned, given the very low incidence of MPS [65], it seems plausible that non-syndromic multiple DCs are much more prevalent in the population than the syndromic ones. In other words, there is a higher chance of the occurrence of multiple DCs in MPS patients, but considering the low numbers of MPS patients in the general population, non-syndromic DCs probably constitute a majority of multiple DCs in the population. This is also supported by the numerous case reports of multiple non-syndromic DCs available in the literature (Supplementary Table S2). It should be, however, emphasized that multiple DCs have to be carefully distinguished from multiple hyperplastic calcifying dental follicles [66]. The cause of the occurrence of multiple and bilateral DCs in otherwise healthy patients is unknown. In one patient with bilateral DCs, a polymorphism of the 1qh + chromosome was described [67]. However, no other study investigated this polymorphism so we are unable to draw any conclusions on whether this might be the reason for multiple non-syndromic DC occurrence.

Besides the well-known mutation in the Patched (*PTCH)* gene, molecular pathways associated with primary cilia might also play a role in DC development [68]. Moreover, age-related events in dental follicles are poorly explored and crucial molecular changes leading to cystic transformation may have a higher probability of occurring in older patients with impacted teeth [69]. These age-related events can explain the higher occurrence of DCs among older patients in our population.

### Multiple OKCs

Papers on multiple OKCs report their occurrence to vary from 4.3 to 15.2% of all patients with OKCs; the result detected in our study, 10.5% (Table 2), is consistent with these findings. However, the proportion of OKC patients with NBCCS was relatively higher (7%) than usually reported. Only three studies reported higher rates of NBCCS patients (Table 2).

**Table 2:** Prevalence of multiple odontogenic keratocysts (OKCs) in different countries

Region	Total number of patients with				Reference
	an OKC	multiple OKCs	non-NBCCS multiple OKCs	NBCCS multiple OKCs	
Georgia, USA	87	6 (6.9%)	5 (5.7%)	1 (1.1%)	Payne 1972 [78]
Indiana, USA	283	20 (7.0%)	10 (3.5%)	10 (3.5%)	Brannon 1976 [79]
Mexico	57	3 (5.3%)	1 (1.8%)	2 (3.5%)	Ledesma-Montes et al. 2000 [80]
Singapore	70	3 (4.3%)	2 (2.8%), both male	1 (1.4%), male	Chow 1998 [81]
South Korea	256	39 (15.2%)	11 (4.3%)	28 (11.0%)	Myoung et al. 2001 [82]
Iran	74	6 (8.1%)	0	6 (8.1%)	Habibi et al. 2007 [83]
South Korea	181	7 (3.9%)	3 (1.7%)	4 (2.2%)	Yang et al. 2011 [84]
India	186	20 (10.8%)	14 (7.5%)	6 (3.2%)	Singh et al. 2013 [85]
Saudi Arabia	75	10 (13.3%)	7 (9.3%)	3 (4.0%)	Bello 2016 [86]
China	455	50 (11.0%)	31 (6.8%)	19 (4.2%)	Fidele et al. 2019 [87]
Italy	113	23 (19.2%)	0	23 (19.2%)	Favia et al. 2022 [88]
Turkey	43	3 (6.9%)	1 (2.3%)	2 (4.7%)	Yilmaz et al. 2022 [89]
India	32	2 (6.3%)	1 (3.1%)	1 (3.1%)	Ac et al. 2023 [90]
Czech Republic	85	9 (10.5%)	3 (3.5%)	6 (7.0%)	Current study

NBCCS – Naevoid Basal Cell Carcinoma Syndrome

Only 3.5% of non-syndromic OKC patients in our cohort developed multiple OKCs. This is about half of the number of non-syndromic multiple OKCs reported by González-Alva (2008), who identified multiple lesions in 24 cases (13.1%) in their group of 183 patients with OKCs [70]. Of those 24 patients, 11 (6%) were associated with NBCCS and the remaining 13 (7.1%) were non-syndromic.

NBCCS is caused mostly by a mutation in the *PTCH* gene, but non-syndromic multiple OKCs are regarded as an incomplete variant of NBCCS [71]. Hence, just like NBCCS, non-syndromic OKCs might also have a familial occurrence [2, 72–74]. While many cases of non-syndromic OKCs have been reported in the literature (Supplementary Table S3), available retrospective studies suggest that NBCCS-associated multiple OKCs are more prevalent than non-syndromic ones (Table 2).

### Multiple OOCs

In our study, only 4 cases of OOCs were retrieved, all in the mandible and without recurrence after enucleation. One of the patients had bilateral synchronous cysts in the posterior area of the mandible. This seemingly high absolute proportion (25%) is, however, probably just a chance finding caused by the very low number of OOCs in our cohort as multiple OOCs generally occur very rarely. Oh et al. (2022) summarized ten reported multiple OOCs in a literature review [29]; later the same year, Ono et al. (2022) published a study on three more cases [30], making up a total of only thirteen cases so far. Most of them had two OOCs, two patients had three OOCs and in one case described by Cheng et al. (2014), the patient had four OOCs [75]. None of the patients were known to have a syndrome or systemic condition. The genetic background of OOCs is still unclear – while Wang et al. (2022) suggested that OOCs don’t harbor the *PTCH* mutation and, therefore, arise due to different reasons than OKCs [76], Ono et al. (2022) reported *PTCH* mutations in all three studied patients with multiple OOCs [30]. This suggests that at least patients with multiple OOCs might have a genetic trait responsible for the multiple occurrence of OOCs.

### Limitations

While this epidemiological study is one of the most comprehensive ones among studies focusing on the occurrence of various types of multiple DOCs and their relation to syndromes, it has some limitations. First, due to its monocentric character and the timeframe limited to approximately 10 years, the number of patients included is lower than in other studies. For this reason, the sample size for subgroups with multiple cysts was lower than in comparable epidemiological studies on the occurrence of DOCs, limiting our ability to perform deeper statistical analyses. For example, the prevalence of multiple OOCs (25%) within the OOC group may be less precise due to the small sample size. Furthermore, the monocentric design means that the study population reflects the demographic and clinical characteristics of a single institution, possibly limiting the generalizability of our findings to other populations or geographic regions. As a retrospective study, our findings are also limited by the quality and completeness of the medical records and histological data available. Our center treats primarily adult patients (although children with larger DOCs are also referred to our department), which may increase the median ages of patients in our study group. Despite these limitations, the study provides valuable insights into the prevalence and characteristics of multiple DOCs. Future multicenter studies with larger sample sizes are recommended to validate these findings and explore additional factors influencing DOC occurrence.

## Conclusion

Multiple DCs and OKCs seem to be generally more common in the Czech population than reported in other populations as shown in Tables 1 and 2. While in the case of DCs, non-syndromic cases of multiple cysts are much more common than syndromic ones, the opposite is true in the case of multiple OKCs as given in Supplementary Table S1 and Table 2. Multiple OOCs are very rare and so far, none of them have been associated with any syndrome. Other rare cases of multiple DOCs, such as multiple COCs, GOCs, or LPCs/BCs are extremely rare. While the study is unique due to its focus on various multiple DOCs and their syndromic relationship, the cohort of patients included is relatively low. Hence, future multicentric investigations with larger cohorts are needed to provide more information on this topic. Regarding syndromes where multiple impacted and supernumerary teeth are concerned (such as Gardner syndrome, Cleidocranial dysplasia, MPS), we suggest that further studies on the prevalence of multiple DOCs in these patients would be helpful. So far, it seems that only patients with MPS are at a higher risk of multiple DCs. In view of that, research focusing on DCs in these patients aiming to investigate possible specific genetic mutations driving cystogenesis would be beneficial. Nevertheless, we conclude that multiple DOCs may account for a syndromic involvement and, therefore, these patients should be always carefully considered for further examination by other specialists or subsequent genetic testing to rule out potential syndromic involvement (e.g. NBCCS or MPS).

## Electronic supplementary material

Below is the link to the electronic supplementary material.

Supplementary Material 1: Table S1. Data of included patients in the study

Supplementary Material 2: Table S2. Cases and case series of non-syndromic multiple dentigerous cysts

Supplementary Material 3: Table S3. Cases and case series of non-syndromic multiple odontogenic keratocysts

## Data Availability

All data generated or analyzed during this study are included in this published article [and its supplementary information files].

## Abbreviations

DOCs: Developmental odontogenic cysts
DCs: Dentigerous cysts
OKCs: Odontogenic Keratocysts
LPC: Lateral periodontal cyst
BC: Botryoid cyst
OOC: Orthokeratinized odontogenic cysts
GOC: Glandular odontogenic cyst
COC: Calcifying odontogenic cyst
MPS: Mucopolysaccharidosis
NBCCS: Naevoid Basal Cell Carcinoma Syndrome
PTCH: Patched gene
