# Supplementary material for: Developmental odontogenic cysts with special focus on the occurrence of multiple cysts and syndromic association: a single-centre cross-sectional study from the Czech Republic
Source: Orphanet J Rare Dis. 2025 Mar 4;20:103. doi: 10.1186/s13023-025-03623-5 (PMC11881262; doi:10.1186/s13023-025-03623-5)
Supplement: Supplementary file 1 — Supplementary Material 1: Table S1. Data of included patients in the study [file 13023_2025_3623_MOESM1_ESM.docx]

**Supplementary Table S1.** Data of included patients in the study

| **Patient No** | **Sex** | **Age (years)** | **Cyst type** | **Location** | **Quadrant** | **NBCCS/multiple non-syndromic** | **No. of cysts** | **Recurrence** | **Treatment** |
| --- | --- | --- | --- | --- | --- | --- | --- | --- | --- |
| 1 | F | 7 | DC | 38 | 3. | 0 | 1 | 0 | exstirpation |
| 2 | M | 9 | DC | 48 | 4. | 0 | 1 | 0 | exstirpation |
| 3 | F | 9 | DC | 45 | 4. | 0 | 1 | 0 | exstirpation |
| 4 | M | 10 | DC | 37 | 3. | 0 | 1 | 0 | exstirpation |
| 5 | F | 10 | DC | 43 | 4. | 0 | 1 | 0 | exstirpation |
| 6 | F | 10 | DC | 35 | 3. | 0 | 1 | 0 | exstirpation |
| 7 | F | 10 | DC | 33 | 3. | 0 | 1 | 0 | exstirpation |
| 8 | M | 11 | DC | 38, 48 | 3., 4. | multiple non-syndromic | 2 | 0 | exstirpation |
| 9 | M | 12 | DC | 13 | 1. | 0 | 1 | 0 | exstirpation |
| 10 | M | 13 | DC | 25 | 2. | 0 | 1 | 0 | exstirpation |
| 11 | M | 13 | DC | 15 | 1. | 0 | 1 | 0 | exstirpation |
| 12 | M | 14 | DC | 37 | 3. | 0 | 1 | 0 | exstirpation |
| 13 | F | 14 | DC | 18, 37, 47 | 1., 3., 4. | multiple non-syndromic | 3 | 0 | exstirpation |
| 14 | F | 14 | DC | 18 | 1. | 0 | 1 | 0 | exstirpation |
| 15 | M | 16 | DC | 38 | 3. | 0 | 1 | 0 | exstirpation |
| 16 | M | 16 | DC | 48 | 4. | 0 | 1 | 0 | exstirpation |
| 17 | M | 16 | DC | 48 | 4. | 0 | 1 | 0 | exstirpation |
| 18 | F | 16 | DC | 28 | 2. | 0 | 1 | 0 | exstirpation |
| 19 | M | 18 | DC | 48 | 4. | 0 | 1 | 0 | exstirpation |
| 20 | F | 18 | DC | Supernumerary | 1. | 0 | 1 | 0 | exstirpation |
| 21 | F | 19 | DC | 38 | 3. | 0 | 1 | 0 | exstirpation |
| 22 | M | 19 | DC | 18 | 1. | 0 | 1 | 0 | exstirpation |
| 23 | M | 20 | DC | 48 | 4. | 0 | 1 | 0 | exstirpation |
| 24 | M | 22 | DC | 38 | 3. | 0 | 1 | 0 | exstirpation |
| 25 | M | 24 | DC | 38 | 3. | 0 | 1 | 0 | exstirpation |
| 26 | M | 24 | DC | 38 | 3. | 0 | 1 | 0 | exstirpation |
| 27 | M | 24 | DC | 38, 48 | 3., 4. | multiple non-syndromic | 2 | 0 | exstirpation |
| 28 | M | 25 | DC | 38 | 3. | 0 | 1 | 0 | exstirpation |
| 29 | M | 25 | DC | 38 | 3. | 0 | 1 | 0 | exstirpation |
| 30 | M | 25 | DC | 48 | 4. | 0 | 1 | 0 | exstirpation |
| 31 | F | 25 | DC | 38, 48 | 3., 4. | multiple non-syndromic | 2 | 0 | exstirpation |
| 32 | M | 26 | DC | 38 | 3. | 0 | 1 | 0 | exstirpation |
| 33 | F | 26 | DC | 38 | 3. | 0 | 1 | 0 | exstirpation |
| 34 | M | 26 | DC | 38 | 3. | 0 | 1 | 0 | exstirpation |
| 35 | M | 26 | DC | 38 | 3. | 0 | 1 | 0 | exstirpation |
| 36 | M | 27 | DC | 38 | 3. | 0 | 1 | 0 | exstirpation |
| 37 | M | 27 | DC | 38 | 3. | 0 | 1 | 0 | exstirpation |
| 38 | F | 27 | DC | 48 | 4. | 0 | 1 | 0 | exstirpation |
| 39 | F | 27 | DC | 38 | 3. | 0 | 1 | 0 | exstirpation |
| 40 | M | 28 | DC | 48 | 4. | 0 | 1 | 0 | exstirpation |
| 41 | F | 28 | DC | 48 | 4. | 0 | 1 | 0 | exstirpation |
| 42 | M | 29 | DC | 48 | 4. | 0 | 1 | 0 | exstirpation |
| 43 | M | 29 | DC | 48 | 4. | 0 | 1 | 0 | exstirpation |
| 44 | M | 29 | DC | 38 | 3. | 0 | 1 | 0 | exstirpation |
| 45 | F | 29 | DC | 38 | 3. | 0 | 1 | 0 | exstirpation |
| 46 | M | 30 | DC | 48 | 4. | 0 | 1 | 0 | exstirpation |
| 47 | M | 30 | DC | 18 | 1. | 0 | 1 | 0 | exstirpation |
| 48 | M | 30 | DC | 48 | 4. | 0 | 1 | 0 | exstirpation |
| 49 | M | 31 | DC | 38 | 3. | 0 | 1 | 0 | exstirpation |
| 50 | M | 33 | DC | 18 | 1. | 0 | 1 | 0 | exstirpation |
| 51 | M | 33 | DC | 38 | 3. | 0 | 1 | 0 | exstirpation |
| 52 | M | 34 | DC | 48 | 4. | 0 | 1 | 0 | exstirpation |
| 53 | M | 34 | DC | 38 | 3. | 0 | 1 | 0 | exstirpation |
| 54 | M | 35 | DC | 48 | 4. | 0 | 1 | 0 | exstirpation |
| 55 | M | 35 | DC | 23 | 2. | 0 | 1 | 0 | exstirpation |
| 56 | F | 36 | DC | 48 | 4. | 0 | 1 | 0 | exstirpation |
| 57 | M | 36 | DC | 38 | 3. | 0 | 1 | 0 | exstirpation |
| 58 | M | 36 | DC | 48 | 4. | 0 | 1 | 0 | exstirpation |
| 59 | F | 36 | DC | 48 | 4. | 0 | 1 | 0 | exstirpation |
| 60 | M | 36 | DC | 48 | 4. | 0 | 1 | 0 | exstirpation |
| 61 | F | 36 | DC | 38 | 3. | 0 | 1 | 0 | exstirpation |
| 62 | M | 36 | DC | 38 | 3. | 0 | 1 | 0 | exstirpation |
| 63 | M | 36 | DC | Multiple impacted teeth | 1., 3., 4. | multiple non-syndromic | 3 | 0 | exstirpation |
| 64 | M | 37 | DC | 33 | 3. | 0 | 1 | 0 | exstirpation |
| 65 | M | 37 | DC | 38 | 3. | 0 | 1 | 0 | exstirpation |
| 66 | M | 37 | DC | Supernumerary | 1. | 0 | 1 | 0 | exstirpation |
| 67 | M | 37 | DC | 28 | 2. | 0 | 1 | 0 | exstirpation |
| 68 | M | 37 | DC | 35 | 3. | 0 | 1 | 0 | exstirpation |
| 69 | M | 37 | DC | 28, 48 | 2., 4. | multiple non-syndromic | 2 | 0 | exstirpation |
| 70 | M | 38 | DC | 38 | 3. | 0 | 1 | 0 | exstirpation |
| 71 | M | 38 | DC | 48 | 4. | 0 | 1 | 0 | exstirpation |
| 72 | M | 39 | DC | 48 | 4. | 0 | 1 | 0 | exstirpation |
| 73 | F | 39 | DC | 13 | 1. | 0 | 1 | 0 | exstirpation |
| 74 | M | 40 | DC | 48 | 4. | 0 | 1 | 0 | exstirpation |
| 75 | F | 40 | DC | 48 | 4. | 0 | 1 | 0 | exstirpation |
| 76 | M | 40 | DC | 38 | 3. | 0 | 1 | 0 | exstirpation |
| 77 | M | 40 | DC | 38, 48 | 3., 4. | multiple non-syndromic | 2 | 0 | exstirpation |
| 78 | F | 40 | DC | 48 | 4. | 0 | 1 | 0 | exstirpation |
| 79 | F | 40 | DC | 38, 48 | 3., 4. | multiple non-syndromic | 2 | 0 | exstirpation |
| 80 | M | 41 | DC | 38 | 3. | 0 | 1 | 0 | exstirpation |
| 81 | M | 41 | DC | 28 | 2. | 0 | 1 | 0 | exstirpation |
| 82 | M | 41 | DC | 38 | 3. | 0 | 1 | 0 | exstirpation |
| 83 | M | 41 | DC | 38 | 3. | 0 | 1 | 0 | exstirpation |
| 84 | F | 42 | DC | 38 | 3. | 0 | 1 | 0 | exstirpation |
| 85 | F | 42 | DC | 28 | 2. | 0 | 1 | 0 | exstirpation |
| 86 | M | 42 | DC | 13 | 1. | 0 | 1 | 0 | exstirpation |
| 87 | M | 42 | DC | 48 | 4. | 0 | 1 | 0 | exstirpation |
| 88 | F | 42 | DC | 18 | 1. | 0 | 1 | 0 | exstirpation |
| 89 | M | 42 | DC | 48 | 4. | 0 | 1 | 0 | exstirpation |
| 90 | M | 42 | DC | 18, 38, 48 | 1., 2., 4. | multiple non-syndromic | 3 | 0 | exstirpation |
| 91 | F | 43 | DC | 38 | 3. | 0 | 1 | 0 | exstirpation |
| 92 | F | 43 | DC | 38 | 3. | 0 | 1 | 0 | exstirpation |
| 93 | M | 43 | DC | 38 | 3. | 0 | 1 | 0 | exstirpation |
| 94 | M | 43 | DC | 43 | 4. | 0 | 1 | 0 | exstirpation |
| 95 | M | 43 | DC | 48 | 4. | 0 | 1 | 0 | exstirpation |
| 96 | F | 44 | DC | 48 | 4. | 0 | 1 | 0 | exstirpation |
| 97 | M | 44 | DC | 48 | 4. | 0 | 1 | 0 | exstirpation |
| 98 | F | 44 | DC | 48 | 4. | 0 | 1 | 0 | exstirpation |
| 99 | M | 44 | DC | 38 | 3. | 0 | 1 | 0 | exstirpation |
| 100 | M | 44 | DC | 38 | 3. | 0 | 1 | 0 | exstirpation |
| 101 | M | 44 | DC | 48 | 4. | 0 | 1 | 0 | exstirpation |
| 102 | M | 44 | DC | 38 | 3. | 0 | 1 | 0 | exstirpation |
| 103 | M | 44 | DC | 48 | 4. | 0 | 1 | 0 | exstirpation |
| 104 | M | 45 | DC | 38 | 3. | 0 | 1 | 0 | exstirpation |
| 105 | M | 45 | DC | 48 | 4. | 0 | 1 | 0 | exstirpation |
| 106 | M | 45 | DC | 13 | 1. | 0 | 1 | 0 | exstirpation |
| 107 | F | 45 | DC | 33 | 3. | 0 | 1 | 0 | exstirpation |
| 108 | F | 45 | DC | 48 | 4. | 0 | 1 | 0 | exstirpation |
| 109 | M | 45 | DC | 38, 48 | 3., 4. | multiple non-syndromic | 2 | 0 | exstirpation |
| 110 | F | 46 | DC | 38 | 3. | 0 | 1 | 0 | exstirpation |
| 111 | M | 46 | DC | 38 | 3. | 0 | 1 | 0 | exstirpation |
| 112 | M | 46 | DC | 38 | 3. | 0 | 1 | 0 | exstirpation |
| 113 | M | 46 | DC | 38 | 3. | 0 | 1 | 0 | exstirpation |
| 114 | F | 46 | DC | 38 | 3. | 0 | 1 | 0 | exstirpation |
| 115 | M | 46 | DC | 48 | 4. | 0 | 1 | 0 | exstirpation |
| 116 | F | 46 | DC | 18 | 1. | 0 | 1 | 0 | exstirpation |
| 117 | M | 46 | DC | Supernumerary | 1.-2. | 0 | 1 | 0 | exstirpation |
| 118 | M | 47 | DC | 35 | 3. | 0 | 1 | 0 | exstirpation |
| 119 | M | 47 | DC | Supernumerary | 4. | 0 | 1 | 0 | exstirpation |
| 120 | M | 47 | DC | 38 | 3. | 0 | 1 | 0 | exstirpation |
| 121 | M | 47 | DC | 38 | 3. | 0 | 1 | 0 | exstirpation |
| 122 | M | 47 | DC | 18 | 1. | 0 | 1 | 0 | exstirpation |
| 123 | M | 48 | DC | 38 | 3. | 0 | 1 | 0 | exstirpation |
| 124 | M | 48 | DC | 38, 48 | 3., 4. | multiple non-syndromic | 2 | 0 | exstirpation |
| 125 | M | 49 | DC | 38 | 3. | 0 | 1 | 0 | exstirpation |
| 126 | F | 49 | DC | 38 | 3. | 0 | 1 | 0 | exstirpation |
| 127 | F | 49 | DC | 48 | 4. | 0 | 1 | 0 | exstirpation |
| 128 | M | 49 | DC | 38 | 3. | 0 | 1 | 0 | exstirpation |
| 129 | M | 49 | DC | 48 | 4. | 0 | 1 | 0 | exstirpation |
| 130 | M | 49 | DC | 38, 48 | 3., 4. | multiple non-syndromic | 2 | 0 | exstirpation |
| 131 | M | 50 | DC | 48 | 4. | 0 | 1 | 0 | exstirpation |
| 132 | M | 50 | DC | 48 | 4. | 0 | 1 | 0 | exstirpation |
| 133 | M | 50 | DC | 38 | 3. | 0 | 1 | 0 | exstirpation |
| 134 | M | 50 | DC | 38, 48 | 3., 4. | multiple non-syndromic | 2 | 0 | exstirpation |
| 135 | M | 51 | DC | 48 | 4. | 0 | 1 | 0 | exstirpation |
| 136 | M | 51 | DC | 35 | 3. | 0 | 1 | 0 | exstirpation |
| 137 | M | 51 | DC | 48 | 4. | 0 | 1 | 0 | exstirpation |
| 138 | M | 51 | DC | 38 | 3. | 0 | 1 | 0 | exstirpation |
| 139 | M | 51 | DC | 48 | 4. | 0 | 1 | 0 | exstirpation |
| 140 | F | 51 | DC | 48 | 4. | 0 | 1 | 0 | exstirpation |
| 141 | F | 52 | DC | 38 | 3. | 0 | 1 | 0 | exstirpation |
| 142 | M | 52 | DC | 28 | 2. | 0 | 1 | 0 | exstirpation |
| 143 | M | 52 | DC | 38 | 3. | 0 | 1 | 0 | exstirpation |
| 144 | M | 52 | DC | 48 | 4. | 0 | 1 | 0 | exstirpation |
| 145 | M | 52 | DC | 48 | 4. | 0 | 1 | 0 | exstirpation |
| 146 | M | 52 | DC | 38 | 3. | 0 | 1 | 0 | exstirpation |
| 147 | M | 52 | DC | 48 | 4. | 0 | 1 | 0 | exstirpation |
| 148 | M | 52 | DC | 48 | 4. | 0 | 1 | 0 | exstirpation |
| 149 | M | 52 | DC | 48 | 4. | 0 | 1 | 0 | exstirpation |
| 150 | F | 52 | DC | 38 | 3. | 0 | 1 | 0 | exstirpation |
| 151 | M | 53 | DC | 38 | 3. | 0 | 1 | 0 | exstirpation |
| 152 | F | 53 | DC | 38 | 3. | 0 | 1 | 0 | exstirpation |
| 153 | F | 53 | DC | 38 | 3. | 0 | 1 | 0 | exstirpation |
| 154 | M | 53 | DC | 38 | 3. | 0 | 1 | 0 | exstirpation |
| 155 | M | 54 | DC | 48 | 4. | 0 | 1 | 0 | exstirpation |
| 156 | M | 54 | DC | 38 | 3. | 0 | 1 | 0 | exstirpation |
| 157 | M | 54 | DC | 48 | 4. | 0 | 1 | 0 | exstirpation |
| 158 | F | 54 | DC | 48 | 4. | 0 | 1 | 0 | exstirpation |
| 159 | M | 54 | DC | 48 | 4. | 0 | 1 | 0 | exstirpation |
| 160 | F | 54 | DC | 48 | 4. | 0 | 1 | 0 | exstirpation |
| 161 | M | 54 | DC | 28 | 2. | 0 | 1 | 0 | exstirpation |
| 162 | M | 54 | DC | 48 | 4. | 0 | 1 | 0 | exstirpation |
| 163 | M | 54 | DC | 38, 48 | 3., 4. | multiple non-syndromic | 2 | 0 | exstirpation |
| 164 | F | 55 | DC | 48 | 4. | 0 | 1 | 0 | exstirpation |
| 165 | M | 55 | DC | 48 | 4. | 0 | 1 | 0 | exstirpation |
| 166 | F | 55 | DC | 38 | 3. | 0 | 1 | 0 | exstirpation |
| 167 | M | 55 | DC | 48 | 4. | 0 | 1 | 0 | exstirpation |
| 168 | M | 56 | DC | 38 | 3. | 0 | 1 | 0 | exstirpation |
| 169 | F | 56 | DC | 38 | 3. | 0 | 1 | 0 | exstirpation |
| 170 | M | 56 | DC | 48 | 4. | 0 | 1 | 0 | exstirpation |
| 171 | M | 56 | DC | 38 | 3. | 0 | 1 | 0 | exstirpation |
| 172 | M | 56 | DC | 38 | 3. | 0 | 1 | 0 | exstirpation |
| 173 | M | 56 | DC | 48 | 4. | 0 | 1 | 0 | exstirpation |
| 174 | M | 56 | DC | 48 | 4. | 0 | 1 | 0 | exstirpation |
| 175 | M | 56 | DC | 48 | 4. | 0 | 1 | 0 | exstirpation |
| 176 | M | 56 | DC | 48 | 4. | 0 | 1 | 0 | exstirpation |
| 177 | F | 56 | DC | 48 | 4. | 0 | 1 | 0 | exstirpation |
| 178 | M | 57 | DC | 48 | 4. | 0 | 1 | 0 | exstirpation |
| 179 | M | 57 | DC | 48 | 4. | 0 | 1 | 0 | exstirpation |
| 180 | M | 57 | DC | 38 | 3. | 0 | 1 | 0 | exstirpation |
| 181 | M | 57 | DC | 48 | 4. | 0 | 1 | 0 | exstirpation |
| 182 | F | 57 | DC | 48 | 4. | 0 | 1 | 0 | exstirpation |
| 183 | F | 57 | DC | 38 | 3. | 0 | 1 | 0 | exstirpation |
| 184 | F | 57 | DC | 38 | 3. | 0 | 1 | 0 | exstirpation |
| 185 | F | 58 | DC | 38 | 3. | 0 | 1 | 0 | exstirpation |
| 186 | M | 58 | DC | 38 | 3. | 0 | 1 | 0 | exstirpation |
| 187 | F | 58 | DC | 38 | 3. | 0 | 1 | 0 | exstirpation |
| 188 | M | 59 | DC | 48 | 4. | 0 | 1 | 0 | exstirpation |
| 189 | F | 59 | DC | 38 | 3. | 0 | 1 | 0 | exstirpation |
| 190 | M | 59 | DC | 48 | 4. | 0 | 1 | 0 | exstirpation |
| 191 | M | 59 | DC | 48 | 4. | 0 | 1 | 0 | exstirpation |
| 192 | M | 59 | DC | 38 | 3. | 0 | 1 | 0 | exstirpation |
| 193 | M | 59 | DC | 48 | 4. | 0 | 1 | 0 | exstirpation |
| 194 | F | 59 | DC | 43 | 4. | 0 | 1 | 0 | exstirpation |
| 195 | M | 59 | DC | 48 | 4. | 0 | 1 | 0 | exstirpation |
| 196 | M | 60 | DC | 38 | 3. | 0 | 1 | 0 | exstirpation |
| 197 | M | 60 | DC | 48 | 4. | 0 | 1 | 0 | exstirpation |
| 198 | M | 60 | DC | 38 | 3. | 0 | 1 | 0 | exstirpation |
| 199 | M | 60 | DC | 48 | 4. | 0 | 1 | 0 | exstirpation |
| 200 | M | 60 | DC | 48 | 4. | 0 | 1 | 0 | exstirpation |
| 201 | F | 60 | DC | 38 | 3. | 0 | 1 | 0 | exstirpation |
| 202 | M | 61 | DC | 38 | 3. | 0 | 1 | 0 | exstirpation |
| 203 | F | 61 | DC | 38 | 3. | 0 | 1 | 0 | exstirpation |
| 204 | M | 61 | DC | 38 | 3. | 0 | 1 | 0 | exstirpation |
| 205 | F | 61 | DC | 48 | 4. | 0 | 1 | 0 | exstirpation |
| 206 | M | 61 | DC | 38 | 3. | 0 | 1 | 0 | exstirpation |
| 207 | F | 61 | DC | 28 | 2. | 0 | 1 | 0 | exstirpation |
| 208 | F | 62 | DC | 38 | 3. | 0 | 1 | 0 | exstirpation |
| 209 | M | 62 | DC | 48 | 4. | 0 | 1 | 0 | exstirpation |
| 210 | M | 62 | DC | 18 | 1. | 0 | 1 | 0 | exstirpation |
| 211 | F | 62 | DC | 48 | 4. | 0 | 1 | 0 | exstirpation |
| 212 | F | 62 | DC | 18 | 1. | 0 | 1 | 0 | exstirpation |
| 213 | M | 63 | DC | 18 | 1. | 0 | 1 | 0 | exstirpation |
| 214 | F | 63 | DC | 48 | 4. | 0 | 1 | 0 | exstirpation |
| 215 | M | 63 | DC | 38 | 3. | 0 | 1 | 0 | exstirpation |
| 216 | M | 63 | DC | 48 | 4. | 0 | 1 | 0 | exstirpation |
| 217 | M | 63 | DC | 38 | 3. | 0 | 1 | 0 | exstirpation |
| 218 | F | 63 | DC | 48 | 4. | 0 | 1 | 0 | exstirpation |
| 219 | F | 63 | DC | 38 | 3. | 0 | 1 | 0 | exstirpation |
| 220 | M | 64 | DC | 38 | 3. | 0 | 1 | 0 | exstirpation |
| 221 | M | 64 | DC | 38 | 3. | 0 | 1 | 0 | exstirpation |
| 222 | F | 64 | DC | 38 | 3. | 0 | 1 | 0 | exstirpation |
| 223 | F | 64 | DC | 48 | 4. | 0 | 1 | 0 | exstirpation |
| 224 | M | 64 | DC | 38, 48 | 3., 4. | multiple non-syndromic | 2 | 0 | exstirpation |
| 225 | F | 65 | DC | 48 | 4. | 0 | 1 | 0 | exstirpation |
| 226 | F | 65 | DC | 28 | 2. | 0 | 1 | 0 | exstirpation |
| 227 | M | 65 | DC | 38 | 3. | 0 | 1 | 0 | exstirpation |
| 228 | M | 65 | DC | 18 | 1. | 0 | 1 | 0 | exstirpation |
| 229 | F | 65 | DC | 48 | 4. | 0 | 1 | 0 | exstirpation |
| 230 | M | 65 | DC | 48 | 4. | 0 | 1 | 0 | exstirpation |
| 231 | M | 65 | DC | 48 | 4. | 0 | 1 | 0 | exstirpation |
| 232 | M | 65 | DC | 38, 48 | 3., 4. | multiple non-syndromic | 2 | 0 | exstirpation |
| 233 | F | 65 | DC | 38, 48 | 3., 4. | multiple non-syndromic | 2 | 0 | exstirpation |
| 234 | M | 66 | DC | 48 | 4. | 0 | 1 | 0 | exstirpation |
| 235 | M | 66 | DC | 48 | 4. | 0 | 1 | 0 | exstirpation |
| 236 | M | 66 | DC | 48 | 4. | 0 | 1 | 0 | exstirpation |
| 237 | M | 66 | DC | 38 | 3. | 0 | 1 | 0 | exstirpation |
| 238 | F | 66 | DC | 38 | 3. | 0 | 1 | 0 | exstirpation |
| 239 | M | 66 | DC | 38 | 3. | 0 | 1 | 0 | exstirpation |
| 240 | M | 66 | DC | 38 | 3. | 0 | 1 | 0 | exstirpation |
| 241 | M | 67 | DC | 38 | 3. | 0 | 1 | 0 | exstirpation |
| 242 | F | 67 | DC | 48 | 4. | 0 | 1 | 0 | exstirpation |
| 243 | M | 67 | DC | 38 | 3. | 0 | 1 | 0 | exstirpation |
| 244 | M | 67 | DC | 38 | 3. | 0 | 1 | 0 | exstirpation |
| 245 | M | 67 | DC | 38 | 3. | 0 | 1 | 0 | exstirpation |
| 246 | M | 67 | DC | 38 | 3. | 0 | 1 | 0 | exstirpation |
| 247 | M | 67 | DC | 38 | 3. | 0 | 1 | 0 | exstirpation |
| 248 | M | 67 | DC | 38 | 3. | 0 | 1 | 0 | exstirpation |
| 249 | F | 67 | DC | 48 | 4. | 0 | 1 | 0 | exstirpation |
| 250 | F | 67 | DC | 38 | 3. | 0 | 1 | 0 | exstirpation |
| 251 | M | 67 | DC | 48 | 4. | 0 | 1 | 0 | exstirpation |
| 252 | F | 67 | DC | 38 | 3. | 0 | 1 | 0 | exstirpation |
| 253 | M | 68 | DC | 48 | 4. | 0 | 1 | 0 | exstirpation |
| 254 | F | 68 | DC | 38 | 3. | 0 | 1 | 0 | exstirpation |
| 255 | M | 68 | DC | 38 | 3. | 0 | 1 | 0 | exstirpation |
| 256 | F | 68 | DC | 38 | 3. | 0 | 1 | 0 | exstirpation |
| 257 | M | 68 | DC | 38 | 3. | 0 | 1 | 0 | exstirpation |
| 258 | M | 68 | DC | 48 | 4. | 0 | 1 | 0 | exstirpation |
| 259 | F | 68 | DC | 38 | 3. | 0 | 1 | 0 | exstirpation |
| 260 | M | 69 | DC | 48 | 4. | 0 | 1 | 0 | exstirpation |
| 261 | M | 69 | DC | 48 | 4. | 0 | 1 | 0 | exstirpation |
| 262 | F | 69 | DC | 38 | 3. | 0 | 1 | 0 | exstirpation |
| 263 | F | 69 | DC | 38 | 3. | 0 | 1 | 0 | exstirpation |
| 264 | M | 70 | DC | 38 | 3. | 0 | 1 | 0 | exstirpation |
| 265 | M | 70 | DC | 38 | 3. | 0 | 1 | 0 | exstirpation |
| 266 | M | 71 | DC | 48 | 4. | 0 | 1 | 0 | exstirpation |
| 267 | F | 71 | DC | 48 | 4. | 0 | 1 | 0 | exstirpation |
| 268 | M | 71 | DC | 38, 48 | 3., 4. | multiple non-syndromic | 2 | 0 | exstirpation |
| 269 | F | 72 | DC | 38 | 3. | 0 | 1 | 0 | exstirpation |
| 270 | M | 72 | DC | 48 | 4. | 0 | 1 | 0 | exstirpation |
| 271 | F | 72 | DC | 48 | 4. | 0 | 1 | 0 | exstirpation |
| 272 | M | 72 | DC | 38, 48 | 3., 4. | multiple non-syndromic | 2 | 0 | exstirpation |
| 273 | F | 73 | DC | 38 | 3. | 0 | 1 | 0 | exstirpation |
| 274 | F | 74 | DC | 48 | 4. | 0 | 1 | 0 | exstirpation |
| 275 | F | 74 | DC | 38 | 3. | 0 | 1 | 0 | exstirpation |
| 276 | F | 75 | DC | 38 | 3. | 0 | 1 | 0 | exstirpation |
| 277 | M | 76 | DC | 48 | 4. | 0 | 1 | 0 | exstirpation |
| 278 | M | 76 | DC | 48 | 4. | 0 | 1 | 0 | exstirpation |
| 279 | M | 76 | DC | 38 | 3. | 0 | 1 | 0 | exstirpation |
| 280 | F | 77 | DC | 38 | 3. | 0 | 1 | 0 | exstirpation |
| 281 | F | 77 | DC | 48 | 4. | 0 | 1 | 0 | exstirpation |
| 282 | F | 77 | DC | 38 | 3. | 0 | 1 | 0 | exstirpation |
| 283 | F | 78 | DC | 38 | 3. | 0 | 1 | 0 | exstirpation |
| 284 | F | 81 | DC | 43 | 4. | 0 | 1 | 0 | exstirpation |
| 285 | M | 82 | DC | 48 | 4. | 0 | 1 | 0 | exstirpation |
| 286 | M | 82 | DC | 38 | 3. | 0 | 1 | 0 | exstirpation |
| 287 | M | 10 | OKC | 18, 13, 28, 34, 44 | 1., 2., 3., 4. | NBCCS | 6 | 1 | exstirpation |
| 288 | F | 12 | OKC | 15, 28, 48, 47 | 1., 2., 4. | NBCCS | 5 | 1 | marsupialisation and exstirpation |
| 289 | F | 12 | OKC | 0 | 4., 3. | NBCCS | 2 | 1 | exstirpation |
| 290 | M | 14 | OKC | 28 | 2. | 0 | 1 | 0 | exstirpation |
| 291 | F | 14 | OKC | 43 | 4. - 3. | 0 | 1 | 1 | exstirpation |
| 292 | F | 14 | OKC | 0 | 3. | 0 | 1 | 1 | exstirpation |
| 293 | F | 14 | OKC | 0 | 1., 3., 4. | NBCCS | 3 | 0 | marsupialisation and exstirpation |
| 294 | M | 15 | OKC | 0 | 3. | 0 | 1 | 0 | exstirpation |
| 295 | F | 17 | OKC | 38 | 3. | 0 | 1 | 0 | exstirpation |
| 296 | M | 18 | OKC | 18, 28, 38, 48 | 1., 2., 3., 4. | NBCCS | 6 | 0 | exstirpation |
| 297 | M | 19 | OKC | 38 | 3. | 0 | 1 | 1 | exstirpation |
| 298 | M | 19 | OKC | 0 | 3. | 0 | 1 | 0 | exstirpation |
| 299 | F | 19 | OKC | 0 | 3. | 0 | 1 | 0 | exstirpation |
| 300 | F | 21 | OKC | 38 | 3. | 0 | 1 | 0 | exstirpation |
| 301 | M | 22 | OKC | 0 | 3. | 0 | 1 | 0 | exstirpation |
| 302 | M | 22 | OKC | 18,17,28 | 1., 2., 4. - 3. | NBCCS | 3 | 1 | exstirpation |
| 303 | M | 23 | OKC | 0 | 4. | 0 | 1 | 1 | exstirpation |
| 304 | M | 24 | OKC | 18 | 1. | 0 | 1 | 0 | exstirpation |
| 305 | M | 24 | OKC | 0 | 3. | 0 | 1 | 0 | exstirpation |
| 306 | M | 25 | OKC | 0 | 4. | 0 | 1 | 0 | exstirpation |
| 307 | F | 26 | OKC | 0 | 3. | 0 | 1 | 0 | exstirpation |
| 308 | F | 26 | OKC | 38, 48 | 4. | multiple non-syndromic | 2 | 0 | exstirpation |
| 309 | M | 26 | OKC | 0 | 4. | 0 | 1 | 1 | exstirpation |
| 310 | F | 27 | OKC | 0 | 3. | 0 | 1 | 0 | exstirpation |
| 311 | F | 27 | OKC | 0 | 4., 3., 2. | multiple non-syndromic | 3 | 1 | exstirpation |
| 312 | M | 28 | OKC | 0 | 4. | 0 | 1 | 0 | exstirpation |
| 313 | F | 29 | OKC | 18 | 1. | 0 | 1 | 0 | exstirpation |
| 314 | M | 29 | OKC | 0 | 3. | 0 | 1 | 0 | exstirpation |
| 315 | M | 30 | OKC | 0 | 3. | 0 | 1 | 0 | exstirpation |
| 316 | M | 30 | OKC | 0 | 3. | 0 | 1 | 0 | exstirpation |
| 317 | F | 31 | OKC | 48 | 4. | 0 | 1 | 0 | exstirpation |
| 318 | F | 32 | OKC | 0 | 4. | 0 | 1 | 0 | exstirpation |
| 319 | F | 33 | OKC | 28 | 2. | 0 | 0 | 0 | exstirpation |
| 320 | F | 33 | OKC | 48 | 4. | 0 | 0 | 0 | exstirpation |
| 321 | F | 33 | OKC | 0 | 2. | 0 | 1 | 0 | exstirpation |
| 322 | M | 35 | OKC | 0 | 4. | 0 | 1 | 0 | exstirpation |
| 323 | M | 35 | OKC | 38 | 3. | 0 | 1 | 0 | exstirpation |
| 324 | M | 35 | OKC | 0 | 2. | 0 | 1 | 0 | exstirpation |
| 325 | M | 37 | OKC | 38 | 3. | 0 | 1 | 0 | exstirpation |
| 326 | M | 38 | OKC | 18, 48 | 1., 4. | multiple non-syndromic | 2 | 0 | exstirpation |
| 327 | F | 38 | OKC | 48 | 4. | 0 | 1 | 0 | exstirpation |
| 328 | M | 38 | OKC | 0 | 4. - 3. | 0 | 1 | 1 | exstirpation |
| 329 | M | 40 | OKC | 0 | 1. | 0 | 1 | 0 | exstirpation |
| 330 | M | 41 | OKC | 0 | 4. | 0 | 1 | 0 | exstirpation |
| 331 | M | 41 | OKC | 0 | 4. | 0 | 1 | 0 | exstirpation |
| 332 | M | 41 | OKC | 0 | 3. | 0 | 1 | 0 | exstirpation |
| 333 | M | 41 | OKC | 0 | 3. | 0 | 1 | 0 | exstirpation |
| 334 | M | 43 | OKC | 0 | 3. | 0 | 1 | 0 | exstirpation |
| 335 | M | 45 | OKC | 0 | 4. - 3. | 0 | 1 | 1 | exstirpation |
| 336 | M | 46 | OKC | 0 | 4. | 0 | 1 | 0 | exstirpation |
| 337 | M | 46 | OKC | 0 | 3. | 0 | 1 | 1 | exstirpation |
| 338 | F | 48 | OKC | 48 | 4. | 0 | 1 | 0 | exstirpation |
| 339 | M | 49 | OKC | 0 | 4. - 3. | 0 | 1 | 0 | marsupialisation |
| 340 | F | 49 | OKC | 0 | 4. | 0 | 1 | 0 | exstirpation |
| 341 | M | 50 | OKC | 0 | 4. | 0 | 1 | 0 | marsupialisation and exstirpation |
| 342 | M | 50 | OKC | 0 | 4. | 0 | 1 | 1 | exstirpation |
| 343 | M | 50 | OKC | 0 | 4. | 0 | 1 | 0 | exstirpation |
| 344 | M | 51 | OKC | 0 | 4. | 0 | 1 | 0 | exstirpation |
| 345 | M | 51 | OKC | 0 | 1. - 2. | 0 | 1 | 0 | exstirpation |
| 346 | M | 52 | OKC | 0 | 3. | 0 | 1 | 0 | exstirpation |
| 347 | M | 52 | OKC | 0 | 3. | 0 | 1 | 0 | exstirpation |
| 348 | F | 53 | OKC | 0 | 3. | 0 | 1 | 0 | exstirpation |
| 349 | F | 54 | OKC | 0 | 2. | 0 | 1 | 0 | exstirpation |
| 350 | M | 54 | OKC | 0 | 4. | 0 | 1 | 0 | exstirpation |
| 351 | M | 56 | OKC | 0 | 3. | 0 | 1 | 0 | marsupialisation and exstirpation |
| 352 | M | 57 | OKC | 0 | 3. | 0 | 1 | 0 | exstirpation |
| 353 | F | 59 | OKC | 0 | 4. | 0 | 1 | 1 | marsupialisation and exstirpation |
| 354 | M | 59 | OKC | 0 | 3. | 0 | 1 | 0 | exstirpation |
| 355 | F | 60 | OKC | 0 | 3. | 0 | 1 | 0 | exstirpation |
| 356 | F | 60 | OKC | 0 | 4. | 0 | 1 | 0 | exstirpation |
| 357 | M | 61 | OKC | 0 | 4. | 0 | 1 | 1 | exstirpation |
| 358 | F | 61 | OKC | 0 | 3. | 0 | 1 | 0 | exstirpation |
| 359 | F | 65 | OKC | 0 | 3. | 0 | 1 | 1 | marsupialisation and exstirpation |
| 360 | M | 66 | OKC | 0 | 3. | 0 | 1 | 0 | exstirpation |
| 361 | F | 67 | OKC | 0 | 4. | 0 | 1 | 0 | exstirpation |
| 362 | M | 67 | OKC | 0 | 4. | 0 | 1 | 1 | exstirpation |
| 363 | M | 67 | OKC | 0 | 3. | 0 | 1 | 0 | exstirpation |
| 364 | M | 68 | OKC | 0 | 4. - 3. | 0 | 1 | 0 | exstirpation |
| 365 | F | 69 | OKC | 0 | 3. | 0 | 1 | 0 | exstirpation |
| 366 | F | 71 | OKC | 38 | 3. | 0 | 1 | 0 | exstirpation |
| 367 | M | 71 | OKC | 0 | 4. | 0 | 1 | 0 | exstirpation |
| 368 | M | 74 | OKC | 13 | 1. | 0 | 1 | 0 | exstirpation |
| 369 | F | 77 | OKC | 0 | 4. | 0 | 1 | 1 | exstirpation |
| 370 | F | 86 | OKC | 0 | 3. | 0 | 2 | 0 | exstirpation |
| 371 | M | 91 | OKC | 0 | 4. - 3. | 0 | 1 | 0 | exstirpation |
| 372 | F | 56 | BC | 0 | 4. | 0 | 1 | 0 | exstirpation |
| 373 | M | 20 | OOC | 0 | 3., 4. | 0 | 2 | 0 | exstirpation |
| 374 | M | 33 | OOC | 48 | 4. | 0 | 1 | 0 | exstirpation |
| 375 | M | 50 | OOC | 0 | 1.-2. | 0 | 1 | 0 | exstirpation |
| 376 | M | 43 | OOC | 0 | 3.-4. | 0 | 1 | 0 | exstirpation |
| 377 | F | 46 | COC | 0 | 4. | 0 | 1 | 0 | exstirpation |

M – male, F – female, DC – dentigerous cyst, OKC – odontogenic keratocyst, BC – botryoid cyst, OOC – orthokeratinizing odontogenic cyst, COC – calcifying odontogenic cyst, NBCCS – naevoid basal cell carcinoma syndrome
