# Supplementary material for: Developmental odontogenic cysts with special focus on the occurrence of multiple cysts and syndromic association: a single-centre cross-sectional study from the Czech Republic
Source: Orphanet J Rare Dis. 2025 Mar 4;20:103. doi: 10.1186/s13023-025-03623-5 (PMC11881262; doi:10.1186/s13023-025-03623-5)
Supplement: Supplementary file 2 — Supplementary Material 2: Table S2. Cases and case series of non-syndromic multiple dentigerous cysts [file 13023_2025_3623_MOESM2_ESM.docx]

**Supplementary Table S2.** Cases and case series of non-syndromic multiple dentigerous cysts

| **Case No** | **Reference*** | **Age (years)** | **Sex** | **Impacted / Unerupted teeth associated with the cyst / Location / Jaw / Region / Quadrant / Odontoma** | **Total number of cysts** | **Cases included in Figure 6** |
| --- | --- | --- | --- | --- | --- | --- |
| 1 | Bull 1920^1^ | 11 | M | 2x Mx, 2x Mn | 4 | + |
| 2 | Sprawson 1922^2^ | 18 | M | 37, 43, 46 | 3 | + |
| 3 | Grellier et al. 1923^3^ | 13 | N/A | 1x Mx, 1x Mn | 2 |  |
| 4 | Benett et al. 1937^4^ | 23 | F | 13, 18, 23, 38, 41 | 5 | + |
| 5 | Seeman 1937^5^ | 16 | M | 18, 17, 28, 38, 37, 48, 47 | 4 | + |
| 6 | Beyrent 1938^6^ | 12 | F | 32, 33, 34, 44 or 45 | 2 | + |
| 7 |  | 12 | M | 13, 23, 2x Mn | 4 | + |
| 8 | Feldman 1938^7^ | 14 | N/A | 48, 1x left Mx | 2 |  |
| 9 | Ivy 1939^8^ | 14 | F | 15, 18, 28, 37, 38, 48 | 6 | + |
| 10 |  | 18 | F | 18, 35, 38, 47 | 4 | + |
| 11 |  | 12 | F | 32, 33, 37, 43, 48 | 5 | + |
| 12 |  | 11 | M | 13, 23, 44 | 3 | + |
| 13 |  | 10 | F | 33, 34 | 2 | + |
| 14 | Haddock 1939^9^ | 14 | M | Multiple teeth | Multiple |  |
| 15 | Uhler 1948^10^ | 27 | F | 38, 48 | 2 | + |
| 16 | Bernick, 1949^11^ | 16 | F | Mx (Canine and incisor) and Mn | N/A |  |
| 17 |  | 36 | M | Mn, bilateral molars | N/A |  |
| 18 |  | 25 | M | Mx and Mn | N/A |  |
| 19 |  | 14 | F | Mx and Mn | N/A |  |
| 20 | Pekarsky, 1950^12^ | 48 | F | Multiple cysts in the Mx and Mn associated with multiple impacted teeth | Multiple |  |
| 21 | Tam 1955^13^ | 7 | M | 36, 46 | 2 | + |
| 22 | Henefer 1964^14^ | 52 | F | 18, 28 | 2 | + |
| 23 | Stanback, 1970^15^ | 9 | M | 38, 48 | 2 | + |
| 24 | Callaghan 1973^16^ | 38 | M | 38, 48 | 2 | + |
| 25 | Shimizu et al. 1974^17^ | 10 | F | 2x Mx, 2x Mn | 4 | + |
| 26 | Sheinkopf 1979^18^ | 60 | F | Multiple cysts | Multiple |  |
| 27 | Burton and Scheffer 1980^19^ | 57 | F | 38, 48 | 2 | + |
| 28 | Swerdloff et al 1980^20^ | 7 | F | 38, 48 | 2 | + |
| 29 | Crinzi 1982^21^ | 15 | F | 38, 48 | 2 | + |
| 30 | Norris et al. 1987^223^ | 7 | F | 13, 23, 33, 43 | 4 | + |
| 31 | McDonnell 1988^23^ | 15 | M | Second premolar and second molar in the Mn | 2 | + |
| 32 | Lustmann and Bodner 1988^24^ | 37 | M | Two impacted supernumerary teeth in the Mx | 2 | + |
| 33 | Eidinger 1989^256^ | 15 | M | 38, 48 | 2 | + |
| 34 | O´Neil et al 1989^26^7 | 5 | M | 38, 48 | 2 | + |
| 35 | Toller et al. 1995^27^ | 13 | M | Molars and canines | ? | + |
| 36 | Banderas et al. 1996^28^ | 38 | M | 38, 48 | 2 | + |
| 37 | Smith 1996^29^ | 35 | F | Mn bilateral | 2 | + |
| 38 | Sands and Tocchio 1998^30^ | 3 | F | 31, 36, 41, 46 | 4 | + |
| 39 | Ko et al. 1999^31^ | 42 | M | 38, 48 | 2 | + |
| 40 | De Biase et al. 2001^32^ | 8 | M | 38, 48 | 2 | + |
| 41 | Simsek et al. 2001^33^ | 45 | M | 38, 48 | 2 | + |
| 42 | Ustuner et al. 2003^34^ | 6 | M | 18, 28 | 2 | + |
| 43 | Batra et al. 2004^35^ | 15 | F | 35, 38, 48 | 3 | + |
| 44 | Tournas et al. 2005^36^ | 4 | F | 2 cysts in the right Mx | 2 | + |
| 45 | Xu et al. 2005^37^ | 15 | M | Multiple in Mx and Mn | Multiple |  |
| 46. | Friestas et al. 2006^38^ | 14 | M | 18, 37 | 2 | + |
| 47 | Lung et al. 2006^39^ | 77 | M | 28, 38, 48 | 3 | + |
| 48 | Mahajan et al. 2006^40^ | 13 | M | 35, 44 | 2 | + |
| 49 | Farahani and Lotfalian 2007^41^ | 37 | M | 13, 23, 32, 33, 43 | 5 | + |
| 50 | Yamalik et al. 2007^42^ | 51 | M | 38, 48 | 2 | + |
| 51 | Maurette et al. 2008^43^ | 7 | M | 36, 46 | 2 | + |
| 52 | Fregnani et al. 2008^44^ | 5 | M | 36, 46 | 2 | + |
| 53 | Carter et al. 2008^45^ | 81 | M | 38, 48 | 2 | + |
| 54 | Iatrou et al. 2009^46^ | 12 | F | 15, 35, 45 | 3 | + |
| 55 |  | N/A | N/A | N/A | N/A |  |
| 56 | Cury et al. 2009^47^ | 5 | M | 36, 46 | 2 | + |
| 57 | Korkmaz and Aral 2009^48^ | 63 | M | 38, 48 | 2 | + |
| 58 | McCrea 2009^49^ | 60 | F | 48, 49 | 2 | + |
| 59 | Ki-Baek et al. 2009^50^ | 11 | M | 13, 45 | 2 | + |
| 60 | Prasad et al. 2010^51^ | 12 | F | 13, 23, 45, 44, 43, 42, 85, 84 | 3 | + |
| 61 | Tikekar et al. 2010^52^ | 11 | M | 35, 45 | 2 | + |
| 62 | Saluja et al. 2010^53^ | 22 | M | 11, 12, 15, 21, 22, 25, 33, 35, 43, 44, 45 | Multiple |  |
| 63 | Grewal and Batra et al. 2010^54^ | 11 | M | 13, 23 | 2 | + |
| 64 | Jung et al. 2010^55^ | 14 | F | Mx molars | 2 | + |
| 65 | Kannan et al. 2010^56^ | 32 | M | 13, 23 | 2 | + |
| 66 | Goyal et al. 2010^57^ | 7 | F | 13, 15, 16, 26 | 2 | + |
| 67 | Prabhakar and Sandhu 2011^58^ | 10 | M | 11, 23 | 2 | + |
| 68 | Reddy et al. 2011^59^ | 11 | F | 34, 35, 44, 45 | 2 | + |
| 69 | Tamgadge et al. 2011^60^ | 10 | M | 15, 23 | 2 | + |
| 70 | Özkan et al. 2011^61^ | 29 | M | 38, 48 | 2 | + |
| 71 | Shirazian and Agha-Hosseini 2011^62^ | 10 | M | 35, 45 | 2 | + |
| 72 | Akay et al. 2011^63^ | 7 | F | 35, 45 | 2 | + |
| 73 |  | 8 | F | 35, 45 | 2 | + |
| 74 |  | 7 | M | 35, 45 | 2 | + |
| 75 |  | 8 | M | Mx and Mn second premolars | N/A |  |
| 76 |  | 9 | F | 13, 23 | 2 | + |
| 77 |  | 8 | F | Mx canines and premolars | N/A |  |
| 78 |  | 9 | M | 35, 45 | 2 | + |
| 79 | Kanth et al. 2011^64^ | 9 | F | 35, 45 | 2 | + |
| 80 | Ishihara et al. 2012^65^ | 13 | M | 35, 38, 45 | 3 | + |
| 81 | Ahmed and Speculand 2012^66^ | 36 | F | 38, 48 | 2 | + |
| 82 | Aher et al. 2013^67^ | 24 | M | 18, 28, 38, 48 | 4 | + |
| 83 | Deshpande and Deshpande 2013^68^ | 9 | F | 35,45 | 2 | + |
| 84 | Cura et al. 2014^69^ | 47 | M | 14, 15, 24, 25, 33, 43 | Multiple |  |
| 85 | Imada et al. 2014^70^ | 42 | F | 38, 48 | 2 | + |
| 86 | Morais et al. 2014^71^ | 15 | M | 37, 47 | 2 | + |
| 87 | Jia and Li 2014^72^ | 61 | F | 38, 48 | 2 | + |
| 88 | Naik et al. 2014^73^ | 25 | M | 38, 48 | 2 | + |
| 89 | Sá Fortes et al. 2014^74^ | 33 | M | 37, 38, 47, 48 | 2 | + |
| 90 | Vasiliu et al. 2014^75^ | 38 | F | 38, 48 | 2 | + |
| 91 | Devi et al. 2015^76^ | 17 | M | 11, 12, 21, 22, 23, 33, 35, 44, 45 | 4 | + |
| 92 | Neto et al. 2015^77^ | 49 | M | 1x Mn, 3x Mx | 2 | + |
| 93 | Hansford et al. 2015^78^ | 6 | F | 13, 14, 15, 23, 24, 25, 33, 34, 35, 43, 44, 45, 47, 37 | Multiple |  |
| 94 | Sanjay et al. 2015^79^ | 24 | F | 33, 43 | 2 | + |
| 95 | Kaushik et al. 2015^80^ | 34 | M | 33, 43 | 2 | + |
| 96 |  | 17 | F | 13, 23 | 2 | + |
| 97 | Jeon et al. 2016^81^ | 15 | M | 18, 28, 38, 48 | 4 | + |
| 98 | Gnanaselvi et al. 2016^82^ | 14 | M | 13, 14, 23, 24, 25, 33, 34, 43, 44 | 4 | + |
| 99 | Sheikhi et al. 2016^83^ | 26 | F | 32, 33, 34, 35, odontoma | 5 | + |
| 100 | Majeti et al. 2017^84^ | 17 | M | 37, 48 | 2 | + |
| 101 | Shruthi et al. 2017^85^ | 15 | M | 38, 48 | 2 | + |
| 102 | Dhupar et al. 2017^86^ | 8 | M | 13, 14, 35 | 2 | + |
| 103 | Raghunandan Iyengar et al. 2017^87^ | 7 | M | 36, 46 | 2 | + |
| 104 | Esmaelizadeh et al. 2017^88^ | 8 | M | 38, 48 | 2 | + |
| 105 | Rodriguez Eunice et al. 2017^89^ | 11 | M | 35, 45 | 2 | + |
| 106 | Yonel et al. 2018^90^ | 42 | M | 38, 48 | 2 | + |
| 107 | Khandeparker 2018^91^ | 10 | M | 13, 15, 23 | 2 | + |
| 108 | Vassiaphan 2018^92^ | 27 | M | 38, 48 | 2 | + |
| 109 | Briguglio et al. 2018^93^ | 9 | M | 36, 46, 47 | 2 | + |
| 110 | Moturi and Kaila 2018^94^ | 19 | F | Multiple | Multiple |  |
| 111 | Gogula et al. 2018^95^ | 40 | M | 38, 48 | 2 | + |
| 112 | Sharma and Chauhan 2019^96^ | 27 | F | 18, 28 | 2 | + |
| 113 | Sindi 2019^97^ | 44 | M | 38, 48 | 2 | + |
| 114 | AlKhudair et al. 2019^98^ | 19 | M | Mx bilateral | 2 | + |
| 115 | Pant et al. 2019^99^ | 10 | M | 13, 23 | 2 | + |
| 116 | De Oliveira et al. 2019^100^ | 14 | M | 23, 37, 47 | 3 | + |
| 117 |  | 14 | M | 37, 47 | 2 | + |
| 118 | Mehdizadeh et al. 2019^101^ | 28 | M | 38, 48 | 2 | + |
| 119 | Oliveira et al. 2020^102^ | 10 | F | 13, 23, 47 | 3 | + |
| 120 | Bergamini et al. 2021^103^ | 39 | M | 28, 38, 48 | 3 | + |
| 121 | Fonseca et al. 2020^104^ | 7 | M | 36, 46 | 2 | + |
| 122 | Santos et al. 2020^105^ | 19 | F | 38, 48 | 2 | + |
| 123 | Yaman et al. 2020^106^ | 60 | M | 18, 13, 23, 38, 48 | 5 | + |
| 124 | Boussouni et al. 2021^107^ | 25 | F | 38, 48 | 2 | + |
| 125 | Keogh et al. 2022^108^ | 12 | M | 37, 47 | 2 | + |
| 126 | Thomaz-De-Aquino et al. 2022^109^ | 10 | N/A | 27, 37, 47 | 3 |  |
| 127 | Arici et al. 2022^110^ | 32 | F | 18, 28 | 2 | + |
| 128 | de Almeida Francisquini et al. 2022^111^ | 11 | F | 13, 23 | 2 | + |
| 129 | Herrero et al. 2022^112^ | 6 | M | 36, 46 | 2 | + |
| 130 | Talha et al. 2022^113^ | 43 | M | 13, 23, 33, 43 | 4 | + |
| 131 | Berberi et al. 2023^114^ | 24 | F | 18, 28 | 2 | + |
| 132 | Urs et al. 2023^115^ | 8 | M | 46, 36 | 2 | + |
| 133 | Genç et al. 2023^116^ | 55 | M | 38, 48 | 2 | + |
| 134 | Kimura et al. 2023^117^ | 41 | F | 37, 38, 47, 48 | 2 | + |
| 135 | Rezende et al. 2023^118^ | 5 | M | Two quadrants | 2 | + |
| 136 |  | 12 | M | Two quadrants | 2 | + |
| 137 | Sugauchi et al. 2023^119^ | 59 | F | 38, 48 | 2 | + |
| 138 | Guan 2023^120^ | 40 | F | 38, 48 | 2 | + |

* Only accessible publications with histologically confirmed dentigerous cysts included

+ Only those case reports and case series which included individual information on the following data (age, sex, precise number of cysts and their location) were included in Figure 6

M – male, F – female, Mx – maxilla, Mn – mandible, N/A – not available or not applicable

**References**

1. Bull FB. Case of Cystic Swellings in the Mouth. Proc R Soc Med. 1920;13:61–3.

2. Sprawson E. Case of Multiple Dentigerous Cysts in the Mandible, and some Remarks on the Pathology of such Cysts. Proc R Soc Med. 1922;15:56–64.

3. Grellier B. Case of Multiple Dentigerous Cysts. Proc R Soc Med. 1923;16:43–4.

4. Bennett PH. Multiple Dentigerous Cysts: Report of Case. The Journal of the American Dental Association and The Dental Cosmos. 1937;24:894–8.

5. Seeman GF. Report of four follicular cysts—Dentigerous. International Journal of Orthodontia and Oral Surgery. 1937;23:1138–40.

6. Beyrent JR. Multiple Dentigerous Cysts in Four Members of One Family. The Journal of the American Dental Association and The Dental Cosmos. 1938;25:623–5.

7. Feldman MH. Follicular cyst of mandible. American Journal of Orthodontics and Oral Surgery. 1938;24:892.

8. Ivy RH. Multiple dentigerous cysts: with special reference to occurrence in siblings. Ann Surg. 1939;109:114–25.

9. Haddock TR. Pseudo-Anodontia Associated With Multiple Dentigerous Cyst Formation: Report of A Case. The Journal of the American Dental Association. 1939;26:606–8.

10. Uhler IV. Bilateral Dentigerous Cysts. The Journal of the American Dental Association. 1948;37:729–30.

11. Bernick S. Dentigerous cysts of the jaw. Oral Surg Oral Med Oral Pathol. 1949;2:914–21.

12. Pekarsky RL. Dentigerous cysts of the jaws with unerupted teeth. Oral Surg Oral Med Oral Pathol. 1950;3:860–7.

13. Tam JC. Bilateral dentigerous cysts involving first molars. J Am Dent Assoc. 1955;50:197–8.

14. Henefer EP. Bilateral dentigerous cysts of the maxilla. Report of a case. Oral Surg Oral Med Oral Pathol. 1964;17:296–8.

15. Stanback JS. The management of bilateral cysts of the mandible. Oral Surg Oral Med Oral Pathol. 1970;30:587–91.

16. Callaghan JH. Bilateral impaction of lower third molars in association with bilateral dentigerous cyst formation. A case report. Glasg Dent J. 1973;4:36–8.

17. Shimizu T, Ohira M, Takahashi T, Shimizu T, Okamoto E. A case of multiple dentigerous cyst. Japanese Journal of Oral and Maxillofacial Surgery. 1974;20:56–8.

18. Sheinkopf DE, Sadowsky D, Seife B. Multiple dentigerous cysts in several facial bones. Oral Surg Oral Med Oral Pathol. 1979;48:492.

19. Burton DJ, Scheffer RB. Serratia infection in a patient with bilateral subcondylar impacted third molars and associated dentigerous cysts: report of case. J Oral Surg. 1980;38:135–8.

20. Swerdloff M, Alexander SA, Ceen RF, Ferguson FS. Bilateral mandibular dentigerous cysts in a seven-year-old child. J Pedod. 1980;5:77–84.

21. Crinzi RA. Bilateral dentigerous cysts of the mandible. Oral Surg Oral Med Oral Pathol. 1982;54:367.

22. Norris LH, Piccoli P, Papageorge MB. Multiple dentigerous cysts of the maxilla and mandible: report of a case. J Oral Maxillofac Surg. 1987;45:694–7.

23. McDonnell DG. Bilateral dentigerous cysts. A case history. J Ir Dent Assoc. 1988;34:63.

24. Lustmann J, Bodner L. Dentigerous cysts associated with supernumerary teeth. Int J Oral Maxillofac Surg. 1988;17:100–2.

25. Eidinger GB. Bilateral dentigerous cysts in the child patient. Report of a case and review of the literature. Univ Tor Dent J. 1989;2:20–3.

26. O’Neil DW, Mosby EL, Lowe JW. Bilateral mandibular dentigerous cysts in a five-year-old child: report of a case. ASDC J Dent Child. 1989;56:382–4.

27. Toller MO, Sipahier M, Acikgoz A. CT display of multiple dentigerous cysts of the mandible: a case report. J Clin Pediatr Dent. 1995;19:135–7.

28. Banderas JA, González M, Ramírez F, Arroyo A. Bilateral mucous cell containing dentigerous cysts of mandibular third molars: report of an unusual case. Arch Med Res. 1996;27:327–9.

29. Smith G. Two dentigerous cysts in the mandible of one patient. Case report. Aust Dent J. 1996;41:291–3.

30. Sands T, Tocchio C. Multiple dentigerous cysts in a child. Oral Health. 1998;88:27–9.

31. Ko KS, Dover DG, Jordan RC. Bilateral dentigerous cysts--report of an unusual case and review of the literature. J Can Dent Assoc. 1999;65:49–51.

32. De Biase A, Ottolenghi L, Polimeni A, Benvenuto A, Lubrano R, Magliocca FM. Bilateral mandibular cysts associated with cyclosporine use: a case report. Pediatr Nephrol. 2001;16:993–5.

33. Şi̇mşek B, Çeti̇ner S, Gülteki̇n E. Nonsyndromatik bilateral dentigerous cyst - a case report. Gazi Üniversitesi Diş Hekimliği Fakültesi Dergisi. 2001;18:83–6.

34. Ustuner E, Fitoz S, Atasoy C, Erden I, Akyar S. Bilateral maxillary dentigerous cysts: a case report. Oral Surg Oral Med Oral Pathol Oral Radiol Endod. 2003;95:632–5.

35. Batra P, Roychoudhury A, Balakrishan P, Parkash H. Bilateral dentigerous cyst associated with polymorphism in chromosome 1qh+. J Clin Pediatr Dent. 2004;28:177–81.

36. Tournas AS, Tewfik MA, Chauvin PJ, Manoukian JJ. Multiple unilateral maxillary dentigerous cysts in a non-syndromic patient: A case report and review of the literature. Int J Ped Otorhinolaryngol Extra. 2006;1:100–6.

37. Xu M, Yang KL, Li XH. Multiple dentigerous cysts of the jaw: a case report. Zhonghua Er Bi Yan Hou Tou Jing Wai Ke Za Zhi. 2005;40:110.

38. Freitas DQ, Tempest LM, Sicoli E, Lopes-Neto FC. Bilateral dentigerous cysts: review of the literature and report of an unusual case. Dentomaxillofac Radiol. 2006;35:464–8.

39. Lung KE, Ganatra S, Robinson CE. Multiple Multilocular Dentigerous Cysts with Intra-osseous and Extra-osseous Third Molar Displacement: A Case Report. Oral Health Group. 2006 [Accessed 2024 Jan 14]. Available from: https://www.oralhealthgroup.com/features/multiple-multilocular-dentigerous-cysts-with-intra-osseous-and-extra-osseous-third-molar-displacemen/

40. Mahajan S, Raj V, Boaz K, George T. Non-syndromic bilateral dentigerous cysts of mandibular premolars: a rare case and review of literature. Hong Kong Dent J. 2006;3:129–33.

41. Farahani SS, Lotfalian M. A pigmented dentigerous cyst in a patient with multiple dentigerous cysts of the jaws: a case report. J Contemp Dent Pract. 2007;8:85–91.

42. Yamalik K, Bozkaya S, Erkmen E, Bariş E. Nonsyndromic Bilateral Mandibular Dentigerous Cysts: Report of a Rare Case. Turkiye Klinikleri J Dental Sci. 2007;13:129–34.

43. Allais de Maurette ME, Maurette OBrien PE, Haiter-Neto F, de Moraes M. Tratamiento de quiste dentígero bilateral mandibular por medio de dos tipos de tratamientos: Relato de caso clínico y comparación entre las técnicas. Acta Odontol Venez. 2007;45:109–12.

44. Fregnani ER, Perez DE da C, de Carvalho PAG, Alves FA. Metachronous bilateral dentigerous cysts associated with permanent first molars. J Dent Child. 2008;75:197–200.

45. Carter TG, Brar PS, Tolas A, Beirne OR. Off-label use of recombinant human bone morphogenetic protein-2 (rhBMP-2) for reconstruction of mandibular bone defects in humans. J Oral Maxillofac Surg. 2008;66:1417–25.

46. Iatrou I, Theologie-Lygidakis N, Leventis M. Intraosseous cystic lesions of the jaws in children: a retrospective analysis of 47 consecutive cases. Oral Surg Oral Med Oral Pathol Oral Radiol Endod. 2009;107:485–92.

47. Cury SEV, Cury MDPN, Cury SEN, Pontes FSC, Pontes HAR, Rodini C, et al. Bilateral dentigerous cyst in a nonsyndromic patient: case report and literature review. J Dent Child. 2009;76:92–6.

48. Korkmaz Y, Aral İ. Mandibulada bilateral dentigeröz kist: vaka raporu. Cumhuriyet Dent J. 2011;12:147–51.

49. McCrea S. Adjacent dentigerous cysts with the ectopic displacement of a third mandibular molar and supernumerary (forth) molar: a rare occurrence. Oral Surg Oral Med Oral Pathol Oral Radiol Endod. 2009;107:e15-20.

50. Ki-Baek K, Seon-Mi K, Kyu-Ho Y, Nam-Ki C. Treatment For Idiopathic Multiple Dentigerous Cysts: Case Report. J Korean Acad Pediatr Dent. 2009;36:270–4.

51. Prasad LK, Chakravarthi PS, Sridhar M, Ramakumar Y, Kattimani V. Nonsyndromic Bilateral Maxillary and Unilateral Mandibular Multiple Dentigerous Cysts in a Young Girl: Report of a Rare Case. Int J Clin Pediatr Dent. 2010;3:219–23.

52. Tikekar S, Degwekar SS, Bhowate RR. Bilateral Dentigerous Cyst: An Unusual Case Report and Review of Literature. Journal of Indian Academy of Oral Medicine and Radiology. 2010;22:116.

53. Saluja JS, Ramakrishnan MJ, Vinit GB, Jaiswara C. Multiple dentigerous cysts in a nonsyndromic minor patient: Report of an unusual case. Natl J Maxillofac Surg. 2010;1:168–72.

54. Grewal H, Batra R. Non Syndromic Bilateral Dentigerous Cysts-A Case Report. Int J Dent Clin. 2010;2:49–51.

55. Jung JH, Kang IG, Cha HE, Kim ST. Bilateral Maxillary Dentigerous Cysts in a Non-Syndromic Patient. Korean J Otorhinolaryngol-Head Neck Surg. 2010;53:57–9.

56. Kannan N, Rajendra P, Sreenivasaul P. Bilateral Maxillary Dentigerous Cysts A Case Report. International Journal of Dental Clinics. 2010;2:28–30.

57. Goyal R, Kumar A, Saxena D, Biswas R. Maxillary sinus swelling in a child: clinical dilemma. BMJ Case Rep. 2010;2010:bcr1220092557.

58. Prabhakar V, Sandhu SV. Nonsyndromic bilateral maxillary dentigerous cysts: Review of literature and report of an unusual case. Int J Ped Otorhinolaryngol Extra. 2011;6:5–8.

59. Reddy PM, Kv NS, Tavane PN, Gupta PK. Non Syndromic Bilateral Dentigerous Cysts: An Unusual Case Report. IJCDS. 2011;2:74–6.

60. Tamgadge A, Tamgadge S, Bhatt D, Bhalerao S, Pereira T, Padhye M. Bilateral dentigerous cyst in a non-syndromic patient: Report of an unusual case with review of the literature. J Oral Maxillofac Pathol. 2011;15:91–5.

61. Özkan A, Okçu KM, Sencimen M, Bayar G, Gülses A, Günhan Ö. Nonsyndromic bilateral mandibular dentigerous cyst: A case report. Gulhane Medical Journal. 2011;53:52–5.

62. Shirazian S, Agha-Hosseini F. Non-syndromic bilateral dentigerous cysts associated with permanent second premolars. Clin Pract. 2011;1:e64.

63. Akay MC, Kaya E, Zeytinoğlu M. Treatment of nonsyndromic dentigerous cysts in primary dentition. Clin Cosmet Investig Dent. 2011;3:17–23.

64. Kanth S, Rajkumar K, Krishnana R, Kumar AR, Malini R, Nandhini G, et al. Bilateral Dentigerous cyst in a nine year old child. SRM Journal of Research in Dental Sciences. 2011;2:76.

65. Ishihara Y, Kamioka H, Takano-Yamamoto T, Yamashiro T. Patient with nonsyndromic bilateral and multiple impacted teeth and dentigerous cysts. Am J Orthod Dentofacial Orthop. 2012;141:228–41.

66. Ahmed NM, Speculand B. Removal of ectopic mandibular third molar teeth: literature review and a report of three cases. Oral Surg. 2012;5:39–44.

67. Aher V, Chander PM, Chikkalingaiah RG, Ali FM. Dentigerous cysts in four quadrants: a rare and first reported case. J Surg Tech Case Rep. 2013;5:21–6.

68. Deshpande A, Deshpande N. Bilateral infected dentigerous cyst in a special child. Journal of Cranio-Maxillary Diseases. 2013;2:54–54.

69. Cura N, Hanttash A, Inceoglu B, Orhan K, Oncul AMT. Dentigerous cysts in four quadrants of a nonsyndromic patient: case report and literature review. Oral Radiol. 2015;31:49–58.

70. Imada TSN, Neto VT, Bernini GF, Silva Santos PS, Rubira-Bullen IRF, Bravo-Calderòn D, et al. Unusual bilateral dentigerous cysts in a nonsyndromic patient assessed by cone beam computed tomography. Contemp Clin Dent. 2014;5:240–2.

71. Morais HHA de, Dias TG de S, Vasconcellos RJ de H, Vasconcelos BC do E, Melo AR, Gondim DA, et al. Bilateral mandibular dentigerous cysts: a case report. RGO, Rev Gaúch Odontol. 2014;62:299–304.

72. Jia S, Li B. Osteosarcoma of the jaws: case report on synchronous multicentric osteosarcomas. J Clin Diagn Res. 2014;8:ZD01-03.

73. Naik NP, Kiran RA, Samata Y, Kumar AV. Non Syndromic, Bilateral, Dentigerous Cysts Associated with Inverted Mandibular Third Molars: A Case Report. Journal of Diagnostics. 2014;1:1–5.

74. Sá Fortes RZ, Júnior VS, Modolo F, Mackowiecky E. Kissing molars: Report of a case. Journal of Oral and Maxillofacial Surgery, Medicine, and Pathology. 2014;26:48–51.

75. Vassiliou LV, Lahiri S, Matthews S n. Non-syndromic bilateral dentigerous cysts with significant root resorption: a case report. Oral Surgery. 2015;8:59–62.

76. Devi P, Thimmarasa VB, Mehrotra V, Agarwal M. Multiple dentigerous cysts: a case report and review. J Maxillofac Oral Surg. 2015;14:47–51.

77. Neto MHDM, Peixoto TS, Alves PM, Filho IJP, Da costa neta MC, De castro gomes DQ. Multiple Dentigerous Cysts in a Nonsyndromic Patient: A Rare Case Report. Oral Surg Oral Med Oral Pathol Oral Radiol. 2015;120:e65.

78. Hansford JT, Kelsch RD, Wiltz M. Multiple Radiolucencies of the Jaws in a 6 Year Old. N Y State Dent J. 2015;81:40–3.

79. Sanjay CJ, David CM, Kaul R, Shilpa PS. Kissing dentigerous cysts involving mandibular canines: report of unusual case with review of literature. J Calif Dent Assoc. 2015;43:29–33.

80. Kaushik A, Chaudhry A, Saluja P, Kumar M, Varshney M. Non-syndromic bilateral dentigerous cysts of maxillary and mandibular canines: A case series and review of literature. Journal of Oral and Maxillofacial Surgery, Medicine, and Pathology. 2015;27:562–6.

81. Jeon JY, Park CJ, Cho SH, Hwang KG. Bilateral dentigerous cysts that involve all four dental quadrants: a case report and literature review. J Korean Assoc Oral Maxillofac Surg. 2016;42:123–6.

82. Gnanaselvi UP, Kamatchi D, Sekar K, Narayanan S. Nonsyndromic multiple dentigerous cyst: A rare clinical presentation. SRM Journal of Research in Dental Sciences. 2016;7:114.

83. Sheikhi M, Samandari MH, Kheir MK, Moaddabi AH. Multiple Dentogerous Cysts With a Complex Odontoma: An Unusual Case Report. Avicenna J Dent Res. 2016;8:2–2.

84. Majeti VD, Srikanth D, Mohan A, Mvs S. Bilateral dentigerous cyst of the mandible - a rare entity, case report and review of literature. International Journal of Recent Scientific Research. 2017;8:19878–81.

85. Shruthi R, Priyanka RB, Ranjeeta Y. Bilateral dentigerous cyst - a rare case report. International Journal of Developmental Research. 2017;7:13400–3.

86. Dhupar A, Yadav S, Dhupar V, Mittal HC, Malik S, Rana P. Bi-maxillary dentigerous cyst in a non-syndromic child - review of literature with a case presentation. J Stomatol Oral Maxillofac Surg. 2017;118:45–8.

87. Raghunandan Iyengar A, Beloor Vasudev SB, Fatima R, Guddannanavar Karibasappa G, Patil S. Bilateral dentigerous cyst involving mandibular first molars- a case report. International Journal of Medical Science and Clinical Invention. 2017;4:2727–30.

88. Esmaelizadeh M, Shokri A, Donyavi Z, Ostovarrad F, Ranjzad H, Yarmohammadi S, et al. Cone Beam Computed Tomography Finding of Unusual Bilateral Dentigerous Cysts in a Nonsyndromic Patient: A Case Report. Avicenna J Dent Res. 2017;9:e38623–e38623.

89. Rodriguez Eunice V, Rivera Daniel Q, Ibarra Paola C, Lara Emilio B, Rocha Fernando T. Bilateral dentigerous cyst treated by marsupialization: A case report. Journal of Case Reports and Images in Dentistry. 2017;3:15–9.

90. Yonel Z, McIntosh AP, Donaldson N, Murphy M, Taneja P. Bilateral dentigerous cysts: an updated literature review and report of a case with associated root resorption. Dent Update. 2018;45:1063–82.

91. Khandeparker RV, Khandeparker PV, Virginkar A, Savant K. Bilateral Maxillary Dentigerous Cysts in a Nonsyndromic Child: A Rare Presentation and Review of the Literature. Case Rep Dent. 2018;2018:7583082.

92. Vasiapphan H, Christopher PJ, Kengasubbiah S, Shenoy V, Kumar S, Paranthaman A. Bilateral Dentigerous Cyst in Impacted Mandibular Third Molars: A Case Report. Cureus. 2018;10:e3691.

93. Briguglio F, Briguglio R, Falcomatà D. An Unusual Multicystic Case in a Non-syndromic Patient: A Case Report. World Journal of Dentistry. 2018;9:423–9.

94. Moturi K, Kaila V. Management of Non-syndromic Multiple Impacted Teeth with Dentigerous Cysts: A Case Report. Cureus. 2018;10:e3323.

95. Gogula S, Nagaraj T, Sumana CK, Nigam H. Bilateral dentigerous cyst - A case report. Journal of Medicine, Radiology, Pathology and Surgery. 2018;5:9–11.

96. Sharma S, Chauhan JS. Bilateral ectopic third molars in maxillary sinus associated with dentigerous cyst-A rare case report. Int J Surg Case Rep. 2019;61:298–301.

97. Sindi AM. Bilateral Mandibular Dentigerous Cysts Presenting as an Incidental Finding: A Case Report. Am J Case Rep. 2019;20:1148–51.

98. AlKhudair B, AlKhatib A, AlAzzeh G, AlMomen A. Bilateral dentigerous cysts and ectopic teeth in the maxillary sinuses: A case report and literature review. Int J Surg Case Rep. 2019;55:117–20.

99. Pant B, Carvalho K, Dhupar A, Spadigam A. Bilateral Nonsyndromic Dentigerous Cyst in a 10-Year-Old Child: A Case Report and Literature Review. Int J Appl Basic Med Res. 2019;9:58–61.

100. de Oliveira RC de, Batista JD, Cardoso SV, Rocha FS. Multiple Dentigerous Cysts: two case reports of nonsyndromic twins. Rev Port Estomatol Med Dent Cir Maxilofac. 2019;60:32–6.

101. Mehdizadeh M, S H, A L. Bilateral Dentigerous Cysts in a Non-Syndromic Patient: Literature Review and Report of a Case. J Islam Dent Assoc Iran. 2019;31:57–63.

102. de Oli̇vei̇ra BC, Fi̇schborn A, Pedroso C, Andrei̇s J, Si̇lvei̇ra C, Guzzoni̇ LF, et al. Multiple Dentigerous Cysts in a Child: A Case Report and Radiographic Follow-up. Cumhuriyet Dent J. 2020;23:136–41.

103. Bergamini ML, Sanches GT, Pina PSS, D’Avila RP, Canto AM do, Ogawa CM, et al. Unusual multiple dentigerous cysts evaluated by cone beam computed tomography: a case report on a non-syndromic patient. Braz J Otorhinolaryngol. 2021;87:110–3.

104. Fonseca MFL, De oliveira EM, Guimarães EP, Pereira AC, Silva AMB, De abreu PTR, et al. Bilateral dentigerous cyst: a rare occurrence. Oral Surg Oral Med Oral Pathol Oral Radiol. 2020;129:e58.

105. Santos AF, Hadad H, Colombo LT, Silva RC da, Poli PP, Junior IRG, et al. Bilateral mandibular dentigerous cyst in non-syndromic patient: technical strategy and literature review. Arch Health Invest. 2020;9:159–63.

106. Yaman D, Akay G, Güngör K. Multiple dentigerous cysts with radiological findings in a non-syndromic patient. J Dent Fac Atatürk Uni. 2020;30:122–5.

107. Boussouni S, Laporte C, Gaudinat M, Margaux Collignon A, Nguyen T, Radoï L. Bilateral mandibular dentigerous cysts in a non-syndromic patient: comprehensive review of the literature and case report. French Journal of Dental Medicine. 2020;1–10.

108. Keogh A, Besi E, Ulhaq A. Multi-disciplinary management of an unusual presentation of bilateral mandibular dentigerous cysts in a non-syndromic child. Oral Surg. 2022;15:583–7.

109. Thomaz-De-Aquino AA, Martins-De-Barros AV, Da Silva Sobrinho AR, Ramos LFS, Araújo FSMDS, De Vasconcelos Carvalho M, et al. Multiple dentigerous cysts in a pediatric patient: case report. Oral Surg Oral Med Oral Pathol Oral Radiol. 2022;134:e171.

110. Arici M, Bayar T, Tas-Ozyurtseven B, Gungormus M. Bilateral ectopic third molars in maxillary sinus associated with dentigerous cyst identified with ophthalmic, nasal and maxillary complication: A rare case report. J Oral Maxillofac Pathol. 2022;26:S84–7.

111. de Almeida Francisquini I, de Lima Medeiros Y, Campos HN, Marlière DAA, Assis NMSP. Orthosurgical Treatment of Impacted Canines with the Presence of Bilateral Dentigerous Cysts: A Case Report with Five-Year Follow-Up. J Maxillofac Oral Surg. 2022;

112. Durán Herrero R, Valcárcel Llerandi J, Rivero Castillo OL, Durán Herrero R, Valcárcel Llerandi J, Rivero Castillo OL. Quiste dentígero bilateral en edad pediátrica. Rev Cubana Pediatr. 2022;94:e1742.

113. Talha A, Yadav H, Adurti A, Upadhayay P, Singh G. Multiple Dentigerous Cysts in A Non-Syndromic Patient- A Rare Occurrence A Case Report. International Journal of All Research Education and Scientific Methods. 2022;10:2630–5.

114. Berberi A, Aoun G, Hjeij B, AboulHosn M, Al Assaad H, Azar E. Bilateral ectopic third molar in the maxillary sinuses associated with dentigerous cyst: a case report. Med Pharm Rep. 2023;96:221–4.

115. Urs AB, Nath Basu S, Singh K, Verma A. Bilateral Dentigerous Cysts of the Permanent Mandibular First Molars Treated with Marsupialization. J Dent Child (Chic). 2023;90:57–61.

116. Genç B, Asadov J, Yalçin BK, Çakarer S. Nonsyndromic Mandibular Bilateral Dentigerous Cyst. Turkiye Klinikleri Dishekimligi Bilimleri Dergisi: Turkiye Klinkeri Journal of Dental Sciences. 2023;29:371–4.

117. Kimura K, Sunada N, Ono Y, Fujii T, Watanabe M, Sakamoto T, et al. A case of bilateral class II kissing molars. Journal of Osaka Dental University. 2023;57:217–22.

118. Rezende DDS da M, Souza LL de, Uchôa DCC, Fernandes LA, Lemos JGR de, Santos-Silva AR, et al. Synchronous jawbone diseases: a multicenter retrospective study. Braz Oral Res. 2023;37:e011.

119. Sugauchi A, Uchihashi T, Yokota Y, Kitaoka Y, Inubushi T, Ogaya Y, et al. Simultaneous Bilateral Dentigerous Cysts at the Mandibular Notch with Ectopic Third Molars: A Case Report. j dent oral disord. 2023;9:1–3.

120. Guan HH. Bilateral dentigerous cysts.Case study, Radiopaedia.org [Accessed 2024 Jan 19]. https://doi.org/10.53347/rID-169788
