# Supplementary material for: Developmental odontogenic cysts with special focus on the occurrence of multiple cysts and syndromic association: a single-centre cross-sectional study from the Czech Republic
Source: Orphanet J Rare Dis. 2025 Mar 4;20:103. doi: 10.1186/s13023-025-03623-5 (PMC11881262; doi:10.1186/s13023-025-03623-5)
Supplement: Supplementary file 3 — Supplementary Material 3: Table S3. Cases and case series of non-syndromic multiple odontogenic keratocysts [file 13023_2025_3623_MOESM3_ESM.docx]

**Supplementary Table S3.** Cases and case series of non-syndromic multiple odontogenic keratocysts

| **Case No** | **Reference*** | **Age (years)** | **Sex** | **Location** | **Number of cysts** | **Number of cysts associated with an impacted tooth** | **Cases included in Figure 6** |
| --- | --- | --- | --- | --- | --- | --- | --- |
| 1 | Yeo and Loh 1989^1^ | 15 | F | Bi Post Mx, Bi Post Mn | 4 | 3 | + |
| 2 | Meara et al. 1996^2^ | N/A | N/A | N/A | N/A | N/A |  |
| 3 | Hebbale et al. 2005^3^ | 60 | M | Bi Post Mn | 2 | 0 | + |
| 4 | Auluck et al. 2006^4^ | 22 | N/A | Bi Post Mx, Bi Post Mn | 4 | 0 |  |
| 5 | Sholapurkar et al. 2008^5^ | 24 | M | Fro Mx, Fro Mn, Bi Post Mn | 4 | 3 | + |
| 6 | Devi et al. 2010^6^ | 32 | M | Bi Post Mn | 2 | N/A | + |
| 7 | Rudagi et al. 2010^7^ | 21 | F | Bi Post Mn | 2 | 2 | + |
| 8 | Parikh 2010^8^ | 19 | M | Fro Mx, Post Mn | 2 | 1 | + |
| 9 | Babu et al. 2011^9^ | 32 | M | Bi Post Mn | 2 | 0 | + |
| 10 | Bartake et al. 2011^10^ | 20 | F | Post Mx, Bi Fro Mn | 3 | 0 | + |
| 11 | Wang et al. 2011^11^ | 29 | F | Post Mx, Post Mn | 2 | 2 | + |
| 12 | Guruprasad and Chauhan 2012^12^ | 16 | M | Post Mx, Fro Mx, Fro Mn, Post Mn | 4 | 4 | + |
| 13 | Haitami et al. 2013^13^ | 54 | M | Multiple | Multiple | 0 |  |
| 14 | Sarkar and Rathod 2013^14^ | 14 | M | Fro Mn, Post Mn | 2 | Multiple | + |
| 15 | Rai et al. 2013^15^ | 14 | M | Fro Mx, Post Mx, Fro Mn, Post Mn | 4 | 4 | + |
| 16 | Karaghi and Kalantri 2013^16^ | 11 | M | Fro Mx, Fro Mn, Bi post Mn | 2 | 2 | + |
| 17 | Kurderkar et al. 2013^17^ | 23 | M | Bi Fro Mx, Post Mn + DC Mx | 3 + 1 dentigerous cyst + 1 untreated cyst | 1 (dentigerous cyst) | + |
| 18 | Nirwan et al. 2013^18^ | 10 | F | Fro Mx, Fro Mn, Post Mn | 3 | 3 | + |
| 19 |  | 13 | M | Fro Mx bilat 2x, Fro Mn and Post Mn bilat | 7 | 2 | + |
| 20 |  | 17 | M | Multiple Mx, Fro Mn, Post Mn bilat | Multiple | 3 |  |
| 21 | Srivatsan et al. 2014^19^ | 22 | M | Bi Post Mn | 2 | 2 | + |
| 22 |  | 15 | F | Bi Post Mn | 2 | 1 | + |
| 23 | Okoje-Adesomoju et al. 2014^20^ | 38 | M | Bi Mx | 2 | N/A | + |
| 24 | Ozkan et al. 2014^21^ | 13 | F | Multiple Mx and Mn | Multiple | Multiple |  |
| 25 | Jeyaraj et al. 2014^22^ | 45 | F | Fro Mn, Post Mn | 2 | 0 | + |
| 26 | Ram et al. 2014^23^ | 16 | F | Bi Post Mn | 2 | 2 | + |
| 27 | Hammannnavar et al. 2014^24^ | 20 | M | Bi Post Mx, Bi Post Mn | 4 | 3 | + |
| 28 | Narsapur et al. 2015^25^ | 32 | M | Fro Mx and Post Mx, Bi Post Mn | 4 | 2 | + |
| 29 | Goyal et al. 2015^26^ | 30 | F | Fro Mn, Bi Post Mn | 6 | 1 | + |
| 30 | Alok et al. 2015^27^ | 24 | M | Bi Post Mn, Bi Fro Mn | 4 | 2 | + |
| 31 | Ashoka 2015^28^ | 10 | F | Fro Mx, Fro Mn | 2 | 8 | + |
| 32 | Ahmed et al. 2015^29^ | 58 | M | Fro Mn, Post Mn | 2 | 0 | + |
| 33 | Chaudhari et al. 2015^30^ | 22 | M | Fro Mx, Post Mx, Bi Post Mn | 4 | 2 | + |
| 34 | Golgire 2016^31^ | 20 | F | Bi Post Mx, Bi Post Mn | 4 | 4 | + |
| 35 | Mohajerani et al. 2016^32^ | 15 | M | Bi Post Mx | 2 | 2 | + |
| 36 | Vasconcelos et al. 2017^33^ | 15 | F | Bi Post Mx, Bi Fro Mn + Mn Post | 5 | 3 | + |
| 37 | Reddy et al. 2016^34^ | 14 | F | Bi Post Mx, Bi Post Mn | 4 | 2 | + |
| 38 | Newaskar et al. 2016^35^ | 21 | M | Bi Post Mx | 2 | 2 | + |
| 39 | Shimada et al. 2016^36^ | 26 | F | Bi Post Mn | 2 | 2 | + |
| 40 |  | 27 | F | Bi Post Mn | 2 | 2 | + |
| 41 | Patil et al. 2017^37^ | 25 | M | Multiple Mn | Multiple | 0 |  |
| 42 | Sohanian et al. 2017^38^ | 37 | M | Multiple Mn, Mx | Multiple | 0 |  |
| 43 |  | 14 | F | Fro Mn, Post Mn | 2 | 1 | + |
| 44 | Marimuthu et al. 2018^39^ | 28 | F | Bi Post Mx, Bi post Mn | 4 | 0 | + |
| 45 | Sundaragiri et al. 2018^40^ | 15 | M | Fro Mx, Bi Post Mx, Bi Post Mn | 5 (4 OKC + 1 OOC) | 2 | + |
| 46 |  | 21 | F | Post Mx, Bi Post Mn, Fro Mn | 4 | 2 | + |
| 47 |  | 25 | M | Fro Mx, Bi Post Mn, Ram Mn | 4 | 1 | + |
| 48 |  | 35 | M | Post Mx, Post Mn | 2 | 0 | + |
| 49 | Jayaram et al. 2018^41^ | 19 | M | Bi post Mn | 3 | 2 | + |
| 50 |  | 14 | M | Post Mx, Bi post Mn | 4 | 0 | + |
| 51-67 | Hwang et al. 2018^42^ | mean patient age was 28.4±20.1 years | 8 x M, 9 x F | Mx, Mn | 77 lesions in total | 31 sites |  |
| 68 | Agrawal et al. 2018^43^ | 13 | F | Fro Mn , Bi post Mn, | 3 | 3 | + |
| 69 | Kavitha et al. 2019^44^ | 18 | F | Bi Post Mx | 2 | 2 | + |
| 70 | Sowmya et al. 2019^45^ | 19 | M | Post Mx, Fro Mn, Post Mn | 3 | 3 | + |
| 71 | Jamwal et al. 2019^46^ | 15 | M | Bi Post Mx, Fro Mn, Post Mn | 4 | 3 | + |
| 72 | Alencar et al. 2020^47^ | 32 | M | Bi Post Mn | 2 | N/A | + |
| 73 | Brechard et al. 2020^48^ | 20 | N/A | Post Mx, Post Mn | 2 | 2 |  |
| 74 | Satheesh et al. 2020^49^ | 35 | M | Bi Post Mn | 2 | 2 | + |
| 75 | Bnag et al. 2020^50^ | 32 | F | Bi Post Mx, Bo Post Mn | 4 | 2 | + |
| 76 | Shaikh et al. 2021^51^ | 25 | M | Bi Post Mn | 2 | 0 | + |
| 77 | Mishra et al. 2021^52^ | 13 | F | Fro Mn, Bi Post Mn | 3 | 2 | + |
| 78 | Al-aroomy 2022^53^ | 34 | M | Fro Mx, Fro Mn | 2 | 1 | + |
| 79 | Janjua et al. 2022^54^ | 9 | F | Fro Mx,  2x Post Mn, Fro Mn | 4 | N/A | + |
| 80 |  | 13 | F | Post Mx, 2x Fro Mn, 2x Post Mn | 5 | N/A | + |
| 81 |  | 24 | F | 2x Post Mx, 2x Fro Mn | 4 | N/A | + |
| 82 |  | 50 | M | Fro Mx, Fro Mn, Post Mn | 3 | N/A | + |
| 83 | Indu et al. 2022^55^ | 23 | F | Bi Post Mn (+ DC in Post Mx) | 3 | 2 | + |
| 84 | Bhopathi et al. 2022^56^ | 25 | M | Bi Post Mx, Fro and Post Mn | 3 | 3 | + |
| 85 |  | 23 | F | Bi Post Mx, Bi Post Mn | 4 | 3 | + |
| 86 | Pramanik 2023^57^ | 38 | F | Post Mn, Fro Mn | 2 | 0 | + |

* Only accessible publications with histologically confirmed odontogenic keratocysts included

+ Only those case reports and case series which included individual information on the following data (age, sex, precise number of cysts and their location) were included in Figure 6

M – male, F – female, Bi – bilateral, Fro – frontal region, Post – posterior region, Mx – maxilla, Mn – mandible, N/A – not availabe / not applicable

**References**

1. Yeo JF, Loh FC. Multiple odontogenic keratocysts of the jaws. Case report. Aust Dent J. 1989;34:503–6.

2. Meara JG, Li KK, Shah SS, Cunningham MJ. Odontogenic keratocysts in the pediatric population. Arch Otolaryngol Head Neck Surg. 1996;122:725–8.

3. Hebbale M, Bagewadi A, Keluskar V, Halli R. Bilateral Odontogenic Keratocyst - A Case Report. Journal of Indian Academy of Oral Medicine and Radiology. 2005;17:24.

4. Auluck A, Suhas S, Pai KM. Multiple odontogenic keratocysts: report of a case. J Can Dent Assoc. 2006;72:651–6.

5. Sholapurkar AA, MallelaVarun R, Pai KM, V G. Non-syndromic multiple odontogenic keratocysts: report of case. Revista de Clinica e Pesquisa Odontologica. 2008;4:193–9.

6. Devi BY, Rakesh N, Nisha VA, Sagar P, Prasad K. Bilateral keratocystic odontogenic tumor of mandible. Indian Journal of Multidisciplinary Dentistry. 2010;1:12.

7. Rudagi BM, Kharkar VR, Kini Y. Multiple odontogenic keratocysts with diverse histologic features in a non-syndromic patient. Pravara Med Rev. 2010;2:35–7.

8. Parikh NR. Nonsyndromic multiple odontogenickeratocysts: Report of case. Journal of Advanced Oral Research. 2010;1:71–4.

9. Babu C, Kumar V, Dawar G. Nonsyndromic Bilateral Keratocystic Odontogenic Tumor: A Rare Case. World Journal of Dentistry. 2011;2:342–5.

10. Bartake A, Shreekanth N, Prabhu S, Gopalkrishnan K. Non-syndromic recurrent multiple odontogenic keratocysts: a case report. J Dent. 2011;8:96–100.

11. Wang X, Lu Y, Shen G, Chen W. One germline mutation of PTCH gene in a Chinese family with non-syndromic keratocystic odontogenic tumours. Int J Oral Maxillofac Surg. 2011;40:829–33.

12. Guruprasad Y, Chauhan DS. Multiple odontogenic keratocysts in a nonsyndromic patient. Journal of Cranio-Maxillary Diseases. 2012;1:36–40.

13. Haitami S, Oulammou H, Yahya IB. Non-syndromic multiple odontogenic keratocysts: an other observation. Med Buccale Chir Buccale. 2013;19:259–62.

14. Sarkar RR, Rathod GP. Bilateral keratocystic odontogenic tumor of mandible – A unique pediatric lesion: Case report and review. Int J Ped Otorhinol Extra. 2013;8:140–3.

15. Rai S, Rana AS, Kalra P, Gupta D, Goel S. Multiple keratocystic odontogenic tumors in a non-syndromic minor patient: Report of an unusual case. Journal of Orofacial Sciences. 2013;5:61.

16. Kargahi N, Kalantari M. Non-syndromic multiple odontogenic keratocyst: a case report. J Dent. 2013;14:151–4.

17. Kurdekar RS, Prakash J, Rana AS, Kalra P. Non-syndromic odontogenic keratocysts: A rare case report. Natl J Maxillofac Surg. 2013;4:90–3.

18. Nirwan A, Wanjari SP, Saikhedkar R, Karun V. Multiple non-syndromic odontogenic keratocysts in three siblings. BMJ Case Rep. 2013;2013:bcr2012007503.

19. Srivatsan KS, Kumar V, Mahendra A, Singh P. Bilateral keratocystic odontogenic tumor: A report of two cases. Natl J Maxillofac Surg. 2014;5:86–9.

20. Okoje-Adesomoju VN, Adisa AO, Gbolahan OO, Olajide MA. Bimaxillary keratocystic odontogenic tumour: a case of diagnostic and therapeutic difficulty. Case Rep Med. 2014;2014:194810.

21. Ozkan L, Aksoy S, Orhan K, Cetiner S, Uyanik LO, Buhara O, et al. Case report: multiple keratocystic odontogenic tumour in a non-syndromal pediatric patient. Eur J Paediatr Dent. 2014;15:241–4.

22. Jeyaraj P, Naresh N, Srinivas V. Case report on multiple keratocystic odontogenic tumors of jaws: Comparison of a non-syndromic case versus a case of Gorlin Goltz Syndrome. Journal of Oral and Maxillofacial Surgery, Medicine, and Pathology. 2014;26:569–75.

23. Ram H, Mohammad S, Husain N, Gupta S, Kumar A. Bilateral odontogenic keratocyst of the mandible. J Maxillofac Oral Surg. 2014;13:341–5.

24. Hammannavar R, Holikatti K, Bassappa S, Shinde N, Reddy M, Chidambaram YS. Multiple, multifocal odontogenic keratocysts in non-syndrome patient: a case-report. Oral Health Dent Manag. 2014;13:189–93.

25. Narsapur SA, Choudhari S, Warad NM, Manjunath S. Non-syndromic multiple odontogenic keratocysts associated with dental anomalies: A report of unusual case and its management. Journal of Indian Academy of Oral Medicine and Radiology. 2015;27:268.

26. Goyal S, Verma P, Ladgotra A, Mehta M, Sandhu HK. Non-Syndromic Multiple Keratocystic Odontogenic Tumor: A Rare Case Report and Review of Literature. Journal of Oral Medicine, Oral Surgery, Oral Pathology and Oral Radiology. 2015;1:179–84.

27. Alok A, Panat SR, Singh ID, Singh S. Non-syndromic multiple keratocyst odontogenic tumor: A rare case report. Journal of Indian Academy of Oral Medicine and Radiology. 2015;27:264.

28. Ashoka CA. Non Syndromic Multiple Keratocystic Odontogenic Tumour Occurring in Both the Jaws: Case report and Review of Literature. IOSR Journal of Dental and Medical Sciences. 2015;14:35–41.

29. Ahmed S, Saeed TB, Ali Z. Non syndromic multiple keratocystic odontogenic tumors in a patient in his late 50’s: a case report. Khyber Medical University Journal. 2015;7:81–3.

30. Chaudhari R, Nagare S, Birangane R, Kharadi U, Khairnar S, Parkarwar P. Non-Syndromic Multiple Odontogenic Keratocyst. 1. 2015;1:106–11.

31. Golgire SM, Varekar AA, Patil A, Shetti SS, Magdum D. Non-Syndromic Multiple Odontogenic Keratocysts: A Case Report and Comparison with Syndromic Multiple Odontogenic Keratocysts. J Clin Case Rep. 2016;6:1–4.

32. Mohajerani H, Pakravan AH, Aghdashi SF, Nahvi G, Motamedian SR. Bilateral Keratocystic Odontogenic Tumor Invading Maxillary Sinuses: A Case Report. Journal of Regeneration, Reconstruction & Restoration (Triple R). 2016;1:90–3.

33. Vasconcelos AC, Castro PH de S, Borges AH, Volpato LER. Removal of Multiple Keratocystic Odontogenic Tumors in a Nonsyndromic Patient. Ann Maxillofac Surg. 2017;7:136–9.

34. Reddy GV, Reddy MH, Komali G, Anusha R, Bhagirath PV, Badam RK, et al. Non-syndromic multiple keratocystic odontogenic tumors: An arduous challenge for oral and maxillofacial specialists. International Journal of Case Reports and Images (IJCRI). 2016;7:360–4.

35. Newaskar V, Verma M, Rajmohan S, Dashore D. KCOT Occurring in Bilateral Maxillary Sinus in Non-Syndromic Patient. J Clin Diagn Res. 2016;10:ZD16-18.

36. Shimada Y, Maruoka Y, Yamaji I, Kawai S. Non-Syndromic Familial Keratocystic Odontogenic Tumour: A Rare Case Report in Japanese Identical Twins. J Clin Diagn Res. 2016;10:ZD28–30.

37. Patil N, Sinha S, Pandit H, Shah V. Multiple Odontogenic Keratocyst In A Non-Syndromic Patient. IOSR Journal of Dental and Medical Sciences. 2017;16:80–4.

38. Sohanian S, Seifi S, Dezfouli MK, Kiani M, Khakbaz O. Non-Syndromic Multiple Keratocystic Odontogenic Tumor: A Case Report. JOA. 2017;2:7–11.

39. Marimuthu V, Shetty UA, Shetty P. Tetrad presentation of non-syndromic odontogenic keratocyst: An uphill diagnostic and therapeutic challenge. Dent Med Probl. 2018;55:447–51.

40. Sundaragiri KS, Saxena S, Sankhla B, Bhargava A. Non syndromic synchronous multiple odontogenic keratocysts in a western Indian population: A series of four cases. J Clin Exp Dent. 2018;10:e831–6.

41. Jayaram V, Jayachandran S, Bhaskar YH. Radiological Perspectives in Non Syndromic Multiple Odontogenic Keratocysts: Report of Two Cases and Review of literature. J Int Med Sci Acad. 2018;31:189–92.

42. Hwang DS, Kim YH, Kim UK, Ryu MH, Kim GC. Retrospective clinical study of multiple keratocystic odontogenic tumors in non-syndromic patients. J Korean Assoc Oral Maxillofac Surg. 2018;44:107–11.

43. Agrawal N, Carnelio S, Radhakrishnan R, Kudva A, Rodrigues G. Synchronous Odontogenic Cysts in Mandible. JCDR. 2018;12:10–2.

44. Kavitha EG, Jayanthi P, Senthilmoorthy, Rathy R, Harish RK, Ameena M. Bilateral Odontogenic Keratocyst in a Nonsyndromic Patient: A Case Report and Review of Literature. Oral and Maxillofacial Pathology Journal. 2019;10:30–4.

45. Sowmya K, Akhila CNV, Ravi Prakash A, Rajini Kanth M. Non-syndromic Multiple Odontogenic Keratocyst In A 19 Year Old Patient. IMPACT : International Journal of Research in Applied, Natural and Social Sciences. 2019;7:35–40.

46. Jamwal SK, Rai KK, Ramakanth CK, Veerbhadrappa UK, Geetha NT. Multiple Odontogenic Keratocysts in Nonsyndromic Patients—A Case Report and Review of Literature. Journal of Contemporary Dentistry. 2019;9:46–52.

47. Alencar MarGMD, Neto OtJosDL, Júnior EZDS, Vasconcelos BCDE, Filho JosRL, Júnior NoVR, et al. Conservative surgical treatment of bilateral odontogenic keratocyst of the mandible: case report. Oral Surg Oral Med Oral Pathol Oral Radiol. 2020;130:e236.

48. Brechard PM, Hervé G, Descroix V, Lescaille G, Guyon A. A non-syndromic case of maxillo-mandibular keratocysts. J Oral Med Oral Surg. 2020;26:8.

49. Satheesh C, Rafeeq M, Ganesan S, Priya U. Non Syndromic Multiple Odontogenic Keratocysts In Mandible: A Case Report. Journal of Research and Advancement in Dentistry. 2020;10:103–8.

50. Bnag K, Shenoi R, Iqbal MA, Jain S. Management of Non Syndromic Multiple Odontogenic Keratocyst - A case report with literature review. International Journal of Dental Science and Innovative Research (IJDSIR). 2020;3:7–14.

51. Shaikh MZ, Basannavar A, Kaushik A, Byakodi S, Sharma AP, Gunjal VS. Multiple Odontogenic Keratocyst In Non-Syndromic Patient– A Rare Case Report. Journal of Interdisciplinary Dental Sciences. 2021;10:21–7.

52. Mishra R, Kandel L, Yadav D, Tripathi S, Karki B, Chaurasia N, et al. Multiple Odontogenic Keratocysts in a Non-syndromic Young Patient: An Unusual Case Report. Journal of Universal College of Medical Sciences. 2021;9:87–90.

53. Al-aroomy LA, Alwadeai MS, Saleh HO, Shindy MI. Non-Syndromic Multiple Odontogenic Keratocysts: A Rare Case Report and Review of Literature. International Journal Dental and Medical Sciences Research. 2022;4:506–12.

54. Janjua OS, Tariq R, Khalid MU, Qureshi SM, Ali K. Presentation and management of syndromic and non-syndromic patients with multiple odontogenic keratocysts. Oral and Maxillofacial Surgery Cases. 2022;8:100253.

55. Indu S, Roy ID, Tomar K, Jakka S, Singh AK. A case of non-syndromic multiple odontogenic keratocyst with isolated dentigerous cyst: A diagnostic conundrum. Journal of Dentistry Defense Section. 2022;16:165.

56. Bhopathi A, Srinivas CN, Mohd YQ, Parushetti A. Nonsyndromic cases of multiple odontogenic keratocyst: Series of two cases. MRIMS Journal of Health Sciences. 2022;10:52.

57. Asykarie INA, Pramanik F. Non-syndromic multiple odontogenic keratocyst finding with Cone-beam Computed Tomography (CBCT): A rare case report. Jurnal Radiologi Dentomaksilofasial Indonesia (JRDI). 2023;7:35–40.
